# Pan-phylum *In Silico* Analyses of Nematode Endocannabinoid Signalling Systems Highlight Novel Opportunities for Parasite Drug Target Discovery

**DOI:** 10.3389/fendo.2022.892758

**Published:** 2022-07-01

**Authors:** Bethany A. Crooks, Darrin Mckenzie, Luke C. Cadd, Ciaran J. McCoy, Paul McVeigh, Nikki J. Marks, Aaron G. Maule, Angela Mousley, Louise E. Atkinson

**Affiliations:** Microbes & Pathogen Biology, The Institute for Global Food Security, School of Biological Sciences, Queen’s University Belfast, Belfast, United Kingdom

**Keywords:** endocannabinoid, endocannabinoid signalling, nematode, parasite, genome, transcriptome, drug target, endocannabinoid receptor

## Abstract

The endocannabinoid signalling (ECS) system is a complex lipid signalling pathway that modulates diverse physiological processes in both vertebrate and invertebrate systems. In nematodes, knowledge of endocannabinoid (EC) biology is derived primarily from the free-living model species *Caenorhabditis elegans*, where ECS has been linked to key aspects of nematode biology. The conservation and complexity of nematode ECS beyond *C. elegans* is largely uncharacterised, undermining the understanding of ECS biology in nematodes including species with key importance to human, veterinary and plant health. In this study we exploited publicly available omics datasets, *in silico* bioinformatics and phylogenetic analyses to examine the presence, conservation and life stage expression profiles of EC-effectors across phylum Nematoda. Our data demonstrate that: (i) ECS is broadly conserved across phylum Nematoda, including in therapeutically and agriculturally relevant species; (ii) EC-effectors appear to display clade and lifestyle-specific conservation patterns; (iii) filarial species possess a reduced EC-effector complement; (iv) there are key differences between nematode and vertebrate EC-effectors; (v) life stage-, tissue- and sex-specific EC-effector expression profiles suggest a role for ECS in therapeutically relevant parasitic nematodes. To our knowledge, this study represents the most comprehensive characterisation of ECS pathways in phylum Nematoda and inform our understanding of nematode ECS complexity. Fundamental knowledge of nematode ECS systems will seed follow-on functional studies in key nematode parasites to underpin novel drug target discovery efforts.

## Introduction

Parasitic nematodes inflict a pervasive burden on human, animal and plant health (1). The rapid escalation of anthelmintic resistance, and an over reliance on a limited number of frontline anthelmintics, threatens the global sustainability of parasite control. The need for identification and validation of novel control strategies and chemotherapies for nematode parasites is urgent and requires a robust understanding of unexploited aspects of nematode biology that may offer a source of novel drug target candidates.

Neuromuscular signalling is the primary target for frontline anthelmintics because of its importance to nematode biology. Despite this, many facets of nematode neurobiology, including endocannabinoid signalling (ECS), remain uncharacterised and unexploited for parasite control. The ECS system is a complex lipid signalling pathway involved in the regulation of synaptic transmission *via* retrograde signalling (2, 3), and has been associated with a broad range of immunological, psychological, developmental, neuronal and metabolic physiologies in humans where it has significant therapeutic appeal (4). While mammalian (5–7) and invertebrate (8–13) ECS pathways have been studied extensively, our knowledge of the presence, structure and function of ECS in nematodes is limited (14–19 20).

In vertebrates, endocannabinoids (ECs) primarily activate the canonical cannabinoid G-protein coupled receptors (GPCRs) CB1 and CB2 (21–23), in addition to several other cannabinoid-associated receptors including, for example, transient receptor potential channels (TRP) (24, 25). In contrast, nematodes do not appear to possess homologs of the mammalian-like EC-GPCRs (CB1 and CB2). Instead, the nematode-specific GPCR NPR-19, has been functionally linked to ECS in *C. elegans* (*Ce*-NPR-19) (17, 19, 26, 27). *Ce*-NPR-19 displays only 23% sequence similarity with mammalian CB1 but possesses 50% of the key amino acids required for EC ligand (*N*-arachidonoylethanolamine; anandamide; AEA) binding (12, 27, 28). In addition, *C. elegans* NPR-32 is also activated in response to AEA (17), indicating that additional EC-GPCRs could also be present in nematodes.

**Figure 1:**
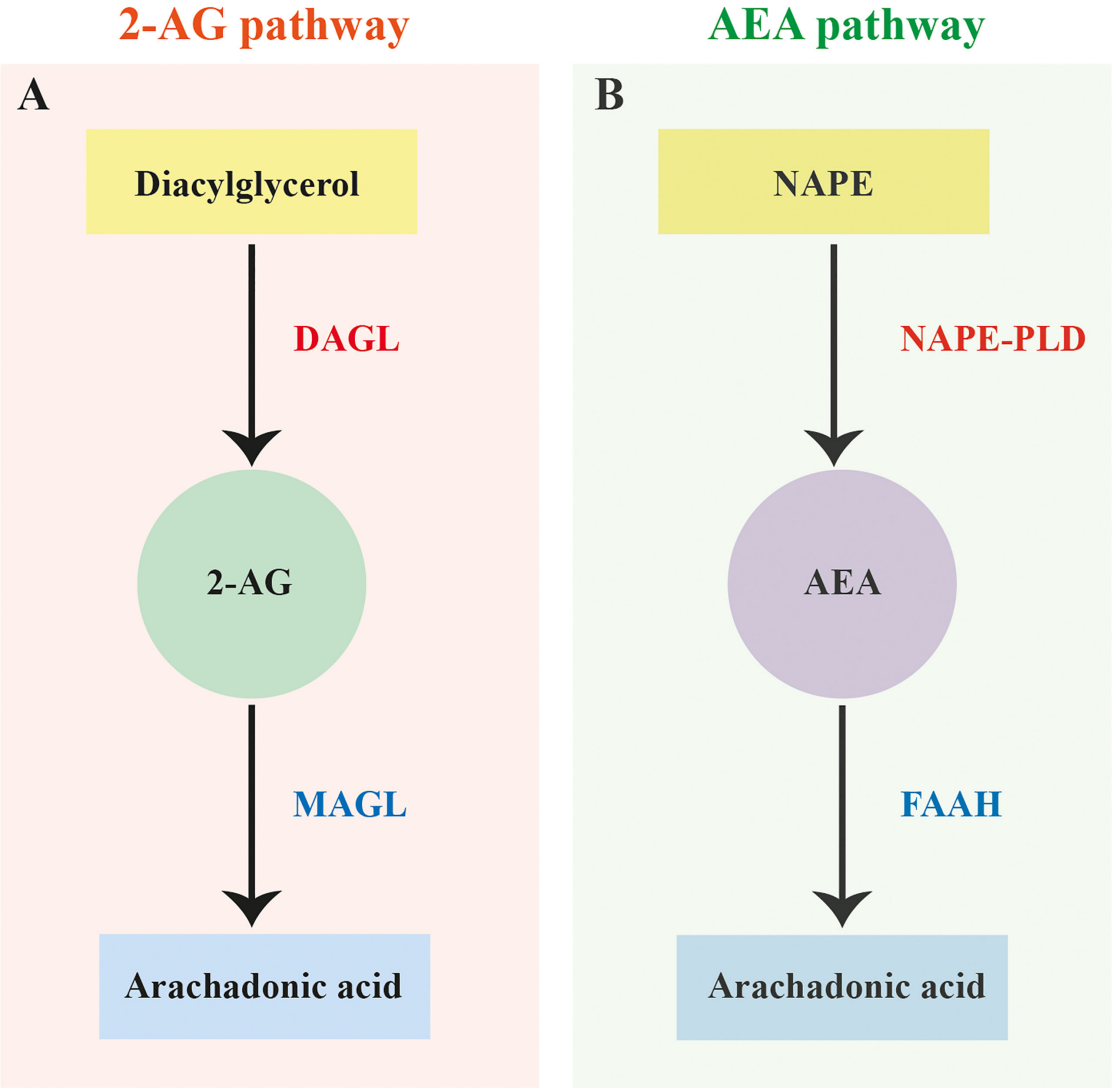
Canonical 2-arachidonoylglycerol and anandamide biosynthesis and degradation pathways. **(A)** Canonical 2-arachidonoylglycerol (2-AG) biosynthesis and degradation pathway based on vertebrates showing the hydrolysis of diacylglycerol by diacylglycerol lipase (DAGL) and degradation of 2-AG by monoacylglycerol lipase (MAGL). **(B)** Canonical anandamide (AEA) biosynthesis and degradation pathway based on vertebrates showing the hydrolysis of N-arachidonyl phosphatidyl ethanol (NAPE) by N-arachidonyl phosphatidyl ethanol-phospholipase D (NAPE-PLD) and degradation of AEA by fatty acid amide hydrolase (FAAH).

In vertebrates the primary EC ligands 2-arachidonoylglycerol (2-AG) and AEA are enzymatically biosynthesised on demand, and subsequently metabolised post receptor activation (see **Figure 1**) (21, 29, 30). 2-AG synthesis occurs *via* the hydrolysis of diacylglycerol by diacylglycerol lipase (DAGL), while degradation can involve several enzymes including monoacylglycerol lipase (MAGL), lysophosphatidylserine lipase alpha/beta-hydrolase domain containing-12 (ABHD-12) , and alpha/beta-hydrolase domain containing 6 (ABHD-6) (see **Figure 1A**) (31–33). AEA is predominantly synthesised *via* the hydrolysis of N-arachidonyl phosphatidyl ethanol (NAPE) by N-arachidonyl phosphatidyl ethanol-phospholipase D (NAPE-PLD) and degraded by fatty acid amide hydrolase (FAAH) (see **Figure 1B**) (34–36). There is also evidence to suggest the presence of multiple alternative pathways for EC-ligand biosynthesis and degradation in mammals involving several alternative enzymes including alpha/beta-hydrolase domain containing-4, N-acyl phospholipase B (ABHD-4), lysophospholipase D (Lyso-PLD) and phospholipase A2 (PLA-2) (36–38).

In nematodes 2-AG and AEA have been identified *via* mass-spectrometry in *C. elegans, Pelodera strongyloides, Caenorhabditis briggsae* and the rodent gastrointestinal nematode *Nippostrongylus brasiliensis* (18, 39). In addition, in *C. elegans* several of the hydrolytic enzymes linked to EC degradation (MAGL and FAAH) have been identified *in silico* (40, 41) and functionally characterised (42 *). Caenorhabditis elegans* ECS has also been associated with a raft of important biological roles (15–17, 19, 27, 42–44).

Information on the presence and function of ECS in parasitic nematodes is limited to a single study that identified ECS enzymes and the putative EC-GPCR NPR-19 *via* bioinformatics in *N. brasiliensis, Ancylostoma duodenale, Ancylostoma celanicum, Necator americanus, Steinernema carpocapsae, Ascaris lumbricoides, Strongyloides ratti, Strongyloides stercoralis, Wuchereria bancrofti* and *Toxocara canis* (18). This work also demonstrated that EC’s may modulate the host immune response during parasite infection and that *N. brasiliensis* produces ECs throughout its lifecycle, most notably in the infective larval stage (18). This strongly supports the hypothesis that parasitic nematodes possess a functional ECS pathway. However, as these observations represent a small subset (6.7%) of available nematode genomes there remains an opportunity to exploit the recent expansion in nematode omics data to characterise the breadth and complexity of the ECS system across phylum Nematoda.

Here, we employed a bioinformatics driven *in silico* pipeline and phylogenetic analyses to identify the presence, and interrogate the conservation and expression profiles of ECS pathway effectors (EC-effectors) in all publicly available nematodes genomes and life stage and tissue-specific transcriptomes. Our data demonstrate that: (i) ECS is broadly conserved across phylum Nematoda, including in therapeutically and agriculturally relevant species; (ii) EC-effectors appear to display clade and lifestyle-specific conservation patterns; (iii) filarial species possess a reduced EC-effector complement; (iv) sequence analyses reveal key differences between nematode and vertebrate EC-effectors; (v) life stage-, tissue- and sex-specific EC-effector expression profiles suggest a role for ECS in therapeutically relevant parasitic nematodes. To our knowledge this study represents the most comprehensive, pan-phylum, analysis of the nematode ECS system, including in species with global therapeutic and agricultural significance. These data will facilitate basic research focused on of the role of EC’s in key aspects of nematode biology e.g. motility, sensory function, feeding, development, and will seed functional genomics studies in tractable parasitic nematodes to inform future novel anthelmintic target discovery pipelines.

## Materials and Methods

### Retrieval of Query Sequences

Query sequences for 70 genes encoding a total of 14 C*. elegans, C. briggsae, Caenorhabditis brenneri, Caenorhabditis japonica* and *Caenorhabditis remanei* EC pathway effectors [seven EC receptors (ECRs) including receptors that have been closely linked to ECS, and seven endocannabinoid enzymes (ECEs)] were obtained from WormBase ParaSite v14 (WBP; https://parasite.wormbase.org) (48) (see **File SI 1**) (18, 27, 43, 45, 46). Note that in the nematode literature, NAPE-PLD orthologs are commonly referred to as NAPE (42, 43, 47) [WormBase Gene IDs; *nape-1* WBGene00021371, *nape-2* WBGene00021370 (48)], consequently we have continued to refer to nematode NAPE-PLD as NAPE in this study for consistency.

### Hidden Markov Model and BLAST Analysis

A Hidden Markov Model (HMM)-based approach has previously been reported for the identification of flatworm GPCRs (49). Predicted protein datasets were downloaded from WBP v14 for all nematodes with publicly available genome data (134 genomes; see **File SI 2**). Predicted protein datasets were concatenated for use as a predicted protein database for HMMERv3.3 HMM-searches. Profile HMMs for nematodes were constructed using predicted protein alignments of all candidate EC-effector protein homologs in *C. elegans, C. briggsae, C. brenneri, C. japonica* and *C. remanei* (see **File SI 1**). Multiple Sequence Alignments (MSAs) were generated using EMBL-EBI Clustal-Omega [https://www.ebi.ac.uk/Tools/msa/clustalo; (50)]. The *hmmsearch* function was employed to identify putative EC proteins within the nematode predicted protein datasets (see **File SI 2**) using default settings. Due to the volume of genomic data generated, the highest confidence hits were selected for each protein based on an inclusion threshold of E-value ≤0.01 and/or a score of ≥150.

Putative EC protein sequences identified *via hmmsearch* were then used as queries in reciprocal BLASTp searches of WBP (https://parasite.wormbase.org/Multi/Tools/Blast; default settings) and NCBI non-redundant (https://blast.ncbi.nlm.nih.gov; default settings) databases. Queries that failed to return a putative ECR or ECE hit within the top 4 BLAST results were excluded from further downstream analyses. All BLASTp searches returning negative hits (hits outside of the outlined inclusion criteria and those that returned no hits) were confirmed negative *via* tBLASTn searches; this approach also mitigated the impact of poor genome quality (false negatives) on our analyses where relevant.

### Post-BLAST Sequence Analysis

Key EC ligand binding residues and functional motifs for mammalian and invertebrate ECRs and ECEs were identified from the published literature (see **File SI 3**) and all positive hits were examined visually for the presence of any key residues/motifs *via* multiple sequence alignments using EMBL-EBI Clustal-Omega [https://www.ebi.ac.uk/Tools/msa/clustalo (50)]. The presence of known protein family or structural domains in ECE BLAST hits was analysed using InterProScan [https:www.ebi.ac.uk/interpro/search/sequence-search, (51)]. Putative ECR hits were analysed for the presence of GPCR transmembrane (TM) domains using EMB TMpred server [https://embnet.vital-it.ch/software/TMPRED_form.html (52)]. Any putative ECE hits which lacked the required family/protein domains for designation as an ECE, or putative ECR sequences which possessed ≤ 3 TM domains, were excluded from further analysis. ECRs and ECEs were analysed for the presence of conservative substitutions of key residues or within binding motifs using WebLogo3 (53, 54) (see **Supplementary Table 2**).

### Phylogenetic Analysis

MUSCLE was used to create multiple sequence alignments (MSAs) of protein sequences for all positive EC protein hits using MEGA X (55). For ECRs, alignments were manually edited to include only TM domains, for ECEs only functional domains were included in analysis. Functional domains for ECEs and TM domains for ECRs were identified *via* the NCBI Conserved Domains Database [CDD; https://www.ncbi.nlm.nih.gov/Structure/cdd/wrpsb.cgi (56)]. Maximum likelihood (ML) phylogenetic trees were constructed using PhyML [http://www.phylogeny.fr (57)] from the domain only MUSCLE MSAs with default parameters and branch support assessment using the approximate likelihood ratio test (aLRT) with “SH-like” parameters. Trees were exported from PhyML in Newick format and were drawn and annotated using the Interactive Tree of Life [iTOL; https://itol.embl.de (58).

### Transcriptome Analysis

180 publicly available transcriptome datasets (145 life stage- and 35 tissue-specific datasets) representing 32 nematode species were analysed in this study. One hundred and fifty publicly available life stage and tissue specific transcriptome datasets representing 27 nematode species were collated from WBP v14 Gene Expression database (48) and published literature (see **File SI 2**). WBP datasets (see **File SI 2**) consisted of metadata, raw counts, transcripts per million (TPM) and DESeq2 differential expression data (in log2foldchange and adjusted p value formats). Data for an additional 34 datasets, representing five species [*Haemonchus contortus, Toxocara canis, Globodera pallida, Strongyloides venezuelensis* and *Strongylodies papillosus*; see **File SI 2**] were identified from published literature, and metadata and raw counts were accessed from NCBI Sequence Read Archive [SRA; www.ncbi.nlm.nih.gov/sra] (59) for analysis. TPM and median TPM data for *H. contortus, T. canis* and *G. pallida* were downloaded using the European Bioinformatics Institute RNAseq-er Application Program Interface (60) (**File SI 2**).

*S. venezuelensis* and *S. papillosus* (raw counts and TPM) data were analysed using an established RNA-Seq pipeline (61). Briefly, raw sequences reads were processed into forward and reverse fastq files using the NCBI SRA Toolkit (62). Reads were then trimmed using Trimmomatic (v0.36; parameters: LEADING:5 TRAILING:5 SLIDINGWINDOW:3:15 MINLEN:34) (63) and sequences below this established inclusion threshold were removed. Corresponding genome assemblies [BioProject accessions; PRJEB530 and PRJEB525, respectively] (64) were downloaded from WormBase ParaSite v14 (48) and reads were mapped to the relevant genome using HISAT2 v2.1.0 (65). Raw gene counts were assigned *via* SubRead v 2.0.1 featureCounts (66). Raw counts of orthologous genes were transformed to TPM and subsequently median TPMs were calculated to represent raw gene expression in the life stages with RNA-Seq data available. An inclusion threshold for expression of 1.5 TPM was applied [TPM thresholds are typically set between 1-2 TPM (67, 68)], any transcripts which failed to meet the threshold for expression were excluded from downstream analysis.

Differential expression data for *H. contortus, T. canis, G. pallida, S. venezuelensis* and *S. papillosus* RNA-Seq data were generated using DESeq2 in the format of log2foldchange and adjusted p values (69, 70). Datasets were then mined for pathway protein gene IDs identified in the HMM searches. Heatmaps displaying log2AverageTPM were generated using the Heatmapper Expression protocol, with an average linkage clustering method and Pearson’s distance measurement method (71).

## Results and Discussion

### Nematodes Possess Homologs of Canonical Endocannabinoid Signalling Pathway Effectors

**Figure 2:**
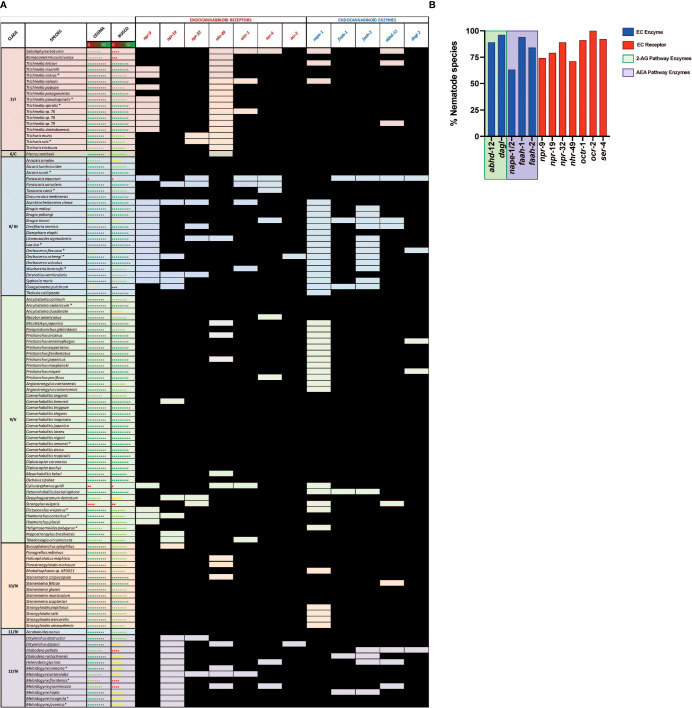
Nematodes possess homologs of canonical endocannabinoid signalling pathway effectors that display broad pan-phylum conservation. **(A)** Pan-phylum conservation of ECS proteins in all nematodes that possess genome data. Black boxes represent the presence of a homolog. Core Eukaryotic Genes Mapping Approach (CEGMA)/Benchmarking Universal Single-Copy Orthologs (BUSCO) scores represent genome quality [data derived from WormbaseParasite, each circle represents 10% increase in genome quality, colours represent scale (red represents lower percentage genome quality, green represents higher percentage genome quality)]. Asterix denotes multiple genomes (*). Nematode genome references listed in **File SI 2**. All homolog gene IDs identified listed in **File SI 3**. Clades based on Holterman and Blaxter classifications where roman numerals represent Blaxter classification and numbers represent Holterman classification (i.e. 2/I denotes Holterman clade 2 and Blaxter classification clade I) (72, 73). **(B)** Bar chart displaying the percentage of nematode species that possess each ECR/ECE.

In this study we mined 133 nematode genomes (representing 109 species, 7 clades and 3 lifestyles) for 13 putative ECS pathway effectors, expanding upon previous studies (18). Our HMM-based *in silico* approach returned a total of 1289 putative ECS effector homologs (ECEs and ECRs; ECRs included some receptors linked to ECS function) (see **Figure 2** and **File SI 3**). The data demonstrate that: (i) ECEs and ECRs display pan-phylum conservation; (ii) representatives of Clades 8 and 12 exhibit the lowest level of EC-effector conservation [clade 8: 68% and 79% of all possible ECEs and ECRs conserved, respectively; clade 12: 83% and 72% of all possible ECEs and ECRs conserved, respectively] and, (iii) free-living and parasitic nematodes display a comparable level of EC-effector conservation [free-living: 91% and 96% of all possible ECEs and ECRs conserved, respectively; parasitic: 83% and 92% of all possible ECEs and ECRs conserved, respectively] (see **Figure 2**). To our knowledge this is the first pan-phylum examination of nematode ECS profiles and represents a comprehensive analysis of ECS pathway conservation in parasitic species that impact human, animal and plant health. Several important points emerge from these data (see below).

### Endocannabinoid Signalling System Effectors Are Broadly Conserved Across Phylum Nematoda

Nematode EC-effectors (ECEs responsible for synthesis and degradation of EC ligands, and putative ECRs) appear to be broadly conserved across phylum Nematoda (**Figure 2A**). ECEs display greater conservation than putative ECRs across all nematodes, with the exception of the ECE *nape*-1/2 which appears to be absent from 37% of nematodes, many of which are representatives of clades 8 and 9 (**Figures 2A, B**). The more conserved profile of ECEs versus ECRs is consistent with the requirement for specific ECEs in the biosynthesis and degradation of EC-ligands and the potential for redundancy among ECRs, which has been documented in other systems (74, 75).

Genes encoding the key ECEs responsible for 2-AG synthesis and metabolism, DAGL-2 and ABHD-12 respectively, are co-conserved in 87% of nematodes (see **Figure 2**), indicating that a significant proportion of nematode species are likely to possess a functional, canonical, 2-AG synthesis and degradation pathway. Most (95%) nematode species examined possess an gene ortholog of the 2-AG biosynthesis enzyme DAGL-2, while the gene encoding ABHD-12, responsible for catalysing 2-AG metabolism is present in 88% of species. *Parascaris equorum* and *Globodera pallida* do not appear to possess either a *dagl-*2 or *abhd-*12 homolog, however their genome quality is lower as indicated by CEGMA/BUSCO scores [see **Figure 2**; (76, 77)].

Genes encoding NAPE and FAAH, the primary enzymes responsible for the synthesis and metabolism of AEA, are co-conserved in 55% of nematode species, suggesting that a significant proportion nematodes have the ability to metabolise AEA (**Figure 2**). This is corroborated by studies that have identified AEA in several nematodes, including *C. elegans* and *N. brasiliensis*, *via* mass-spectrometry (18, 39). While 88% of species encode at least one FAAH homolog, the NAPE-encoding gene is conserved in only 63% of nematodes (either *nape*-1 or -2). Notably, many of the species that lack a *nape* homolog are filarial nematodes, *Strongyloides* species, or members of the Diplogasteroidea superfamily (**Figure 2**). Whilst the absence of *nape* in some species may be explained by genome quality, in other species with robust genome data an alternative AEA synthesis pathway may exist (see below). Previous studies have demonstrated the presence of two, functionally divergent, *nape* orthologs in *C. elegans* that occupy adjacent genomic positions (*nape*-1, IV:3739520.3740880; *nape*-2, IV:3735470.3738925; [WormBase; (78)], share 73% sequence identity, and display complete conservation of the NAPE-PLD signature sequence (43). Our pan-phylum analysis confirms that *C. elegans* is the only nematode species that possesses two distinct NAPE-encoding genes. In all other species that encode NAPE the same gene ID was returned for both the *nape*-1 and *nape*-2 BLASTp searches (one positive *nape* hit was considered a positive return for both *nape*-1 and *nape*-2 and was designated *nape*-1/2; see **Figure 2** and **File SI 3**). The presence of a single *nape-*1/2 in all of the nematodes examined, including other *Caenorhabditis* species, suggests that *nape*-1 and -2 may have arisen as a result of a relatively recent gene duplication event in *C. elegans* (43).

Of the seven putative ECRs included in this study NPR-19 and NPR-32 have been most closely linked to ECS in nematodes (16, 17, 27). Our *in silico* analysis reveals that 79% of the nematode species investigated in this study possess NPR-19 and 89% possess NPR-32, underscoring their putative importance to nematode biology. In addition to NPR-19 and -32, OCR-2 an ortholog of the human transient receptor potential vanilloid channel (TRPV) (79), has also been closely linked to ECS (25). OCR-2 regulates signal transmission and thus modulates several *C. elegans* behaviours (19, 80). It is interesting to note that 100% of nematode species examined in this study possess a gene encoding OCR-2, also suggesting a significant role in nematode biology. NPR-9 has been implicated in locomotion, regulation of innate immune responses, roaming and foraging behaviours in *C. elegans* (81–83), while GPR-55, the human ortholog of nematode NPR-9, is known to interact with human CB1 and CB2 to form functionally important heteromers (84, 85). The lower level of *npr*-9 conservation across phylum Nematoda revealed here (*npr*-9 conserved in 60% of species examined; **Figure 2**) may indicate a less conserved functional role for this putative ECR in nematodes.

### Nematode EC-Effector Conservation Profiles Display Clade Specific Trends

Our data demonstrate distinct conservation patterns of EC-effectors across nematode clades. Whilst clade 9 and 10 nematodes exhibit the highest degree of EC-effector conservation, clade 8 species display the most reduced complements (**Figure 2**). This could be, in part, explained by lower CEGMA/BUSCO scores for clade 8 genomes, such that the profiles presented here may not be a true representation of EC-effector complements in this clade. Variable genome quality is an inherent caveat to *in silico* approaches such as those employed in this study however, the use of tBLASTn for all negative BLASTp returns can help to mitigate this in addition to continued improvements in genome quality (86).

For other clades there appear to be ECR specific trends. For example, *npr-*32 is broadly conserved across all clades, pointing towards a more conserved function for this receptor pan-phylum. Other ECRs display more clade-specific, restricted profiles, including *nhr*-49 which appears to be entirely absent from clade 2 (**Figure 2A**). Clade 2 nematode genome assemblies are high quality [as indicated by CEGMA/BUSCO scores; (48)] and likely provide a true reflection of the *nhr*-49 profile. Notably, *npr-19* is also entirely absent from clade 12 species, indicating that alternative ECRs may contribute to the ECS pathway in these nematodes. Indeed, clade 12 nematodes exhibit broad conservation of other putative ECRs (e.g. *npr-*9, -32, *nhr-*49, *octr-*1, *ser-*4, *ocr-*2; see **Figure 2A**).

### Filarids Possess Distinctive EC-Effector Profiles

Whilst there appear to be limited differences between free-living and parasitic nematode EC-effector profiles in general [96% ECR conservation in free-living nematodes (FL) vs 92% in parasitic species; 91% ECE conservation in free-living nematodes vs 83% in parasitic species], filarial nematodes display distinct EC-effector gaps. For example, filarial species completely lack genes encoding NPR-9 and NAPE-1/2, and have a significantly reduced FAAH-2 encoding gene profile (**Figure 2A**). The absence of NAPE-1/2 points towards the presence of an alternative AEA synthesis pathway in filarids, which may also be the case for other species e.g. *Pristionchus* spp. and *Strongyloides* spp. that likewise lack NAPE-1/2 encoding genes (see below; **Figure 2A**).

### Nematodes That Lack NAPE-1/2 Possess Putative Alternative AEA Synthesis Enzymes

**Figure 3:**
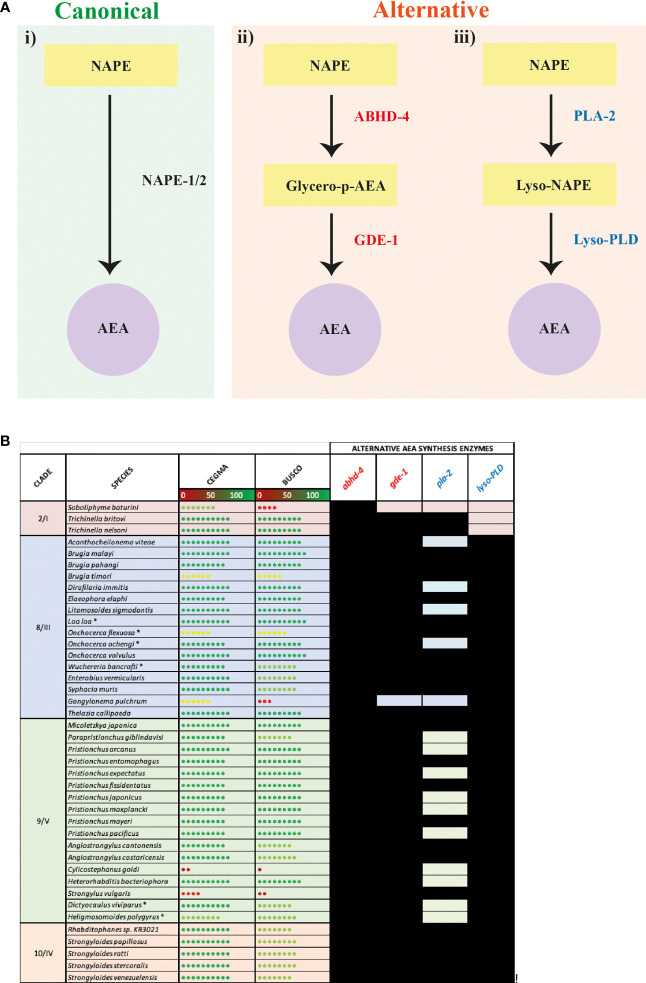
Nematode species lacking the anandamide (AEA) synthesis enzyme N-arachidonyl phosphatidyl ethanol (NAPE) possess putative alternative AEA synthesis enzymes. **(A)** Diagram showing canonical (i) AEA synthesis pathway alongside two alternative synthesis pathways: (ii) hydrolysis of NAPE by Abhydrolase Domain Containing 4 (ABHD-4) forming the intermediate glycerophosphoanandamide (Glycero-p-AEA) and hydrolysis by glycerophosphodiester phosphodiesterase 1 (GDE-1) to synthesise AEA, and (iii) hydrolysis of NAPE by phospholipase A2 (PLA-2) to form the intermediate N-acyl-1-acyl-lyso-PE (lyso-NAPE) and hydrolysis by lysophospholipase D (Lyso-PLD) to synthesise AEA. **(B)** Conservation of genes encoding the alternative AEA synthesis enzymes ABHD-4, GDE-1, PLA-2, Lyso-PLD is shown in all nematodes that lack NAPE. Black boxes represent the presence of a homolog. Core Eukaryotic Genes Mapping Approach (CEGMA)/Benchmarking Universal Single-Copy Orthologs (BUSCO) scores represent genome quality [data derived from WormbaseParasite, each circle represents 10% increase in genome quality, colours represent scale (red represents lower percentage genome quality, green represents higher percentage genome quality)]. Asterix denotes multiple genomes (*). Nematode genome references listed in **File SI 2**. All homolog gene IDs identified listed in **File SI 3**. Clades based on Holterman and Blaxter classifications where roman numerals represent Blaxter classification and numbers represent Holterman classification (i.e. 2/I denotes Holterman clade 2 and Blaxter classification clade I) (72, 73).

Our data indicate that 41 nematode species lack genes encoding the nematode NAPE-PLD ortholog NAPE-1/2, the enzyme primarily responsible for AEA synthesis (**Figure 2A**) (34). In vertebrates, two additional pathways have been implicated in the synthesis of AEA: (i) hydrolysis of NAPE by ABHD-4 forming the intermediate glycerophosphoanandamide (Glycero-p-AEA) which, following further hydrolysis by glycerophosphodiester phosphodiesterase 1 (GDE-1), results in the formation of AEA (see **Figure 3A ii**); (ii) hydrolysis of NAPE by phospholipase A2 (PLA-2) to form the intermediate N-acyl-1-acyl-lyso-PE (lyso-NAPE) and subsequently, following hydrolysis by lysophospholipase D (Lyso-PLD), results in the formation of AEA (see **Figure 3A iii**) (87–90). To determine if alternative AEA synthesis pathways exist in nematodes that appear to lack the classical NAPE-1/2 AEA biosynthesis pathway, we mined all available genome data for *abhd-*4, *gde-*1, *pla-*2 and *lyso-PLD*.

Our data reveal that 39 of the 41 species that lack NAPE-1/2 possess at least one putative alternative AEA synthesis pathway (see **Figure 3B**, **File SI 3**). 95% of these species possess both *abhd*-4 and *gde-*1 (alternative pathway shown in **Figure 3A ii**), while 56% encode both PLA-2 and Lyso-PLD (alternative pathway shown in **Figure 3A iii**)*;* 56% of nematodes encode the enzymes for both alternative AEA synthesis pathways. Therefore these data suggest that nematodes which lack the classical NAPE-1/2 biosynthesis pathway predominantly synthesise AEA *via* ABHD-4 and GDE-1. However, within clades 8, 9 and 10 there are examples of species which may have the ability to synthesise AEA *via* either alternative pathway e.g. *Strongyloides* species and several filarial nematodes (**Figure 3B**).

Interestingly in mammals, in addition to PLA-2, other enzymes have been linked to the synthesis of lyso-NAPE (38), for example, ABHD-4 can remove an acyl group from NAPE resulting in the creation of lyso-NAPE (89). Therefore the presence of *abhd-4* in 100% of the nematodes investigated in this study suggests that some nematodes that possess *lyso-PLD* but lack *pla*-2 could compensate by employing ABHD-4 in the synthesis of lyso-NAPE.

Interestingly, it appears that a subset of the species that do encode NAPE-1/2 (20 key species selected for their relevance to human, veterinary or plant health and broad clade representation), also encode all of the alternative AEA synthesis enzymes (*abhd-*4, *gde-*1, *pla-*2 and *lyso-PLD*), with the exception of *Trichuris muris* which appears to lack *pla-2* (see **File SI 4**). It is unclear whether NAPE-1/2 is indeed the predominant AEA synthesis pathway in these species or, if the alternative pathways also contribute to AEA production.

*In silico* evidence for the presence of putative, alternative, AEA biosynthesis enzymes in a range of therapeutically relevant nematodes strongly suggests that NAPE-1/2 independent pathways may contribute to AEA synthesis in these species. Further analysis, including mass spectrometry to isolate AEA, will begin to unravel the importance of alternative AEA biosynthesis pathways in these nematodes.

### Nematode FAAH Homologs Display Conservative Substitutions in a Key AEA Binding Site

**Figure 4:**
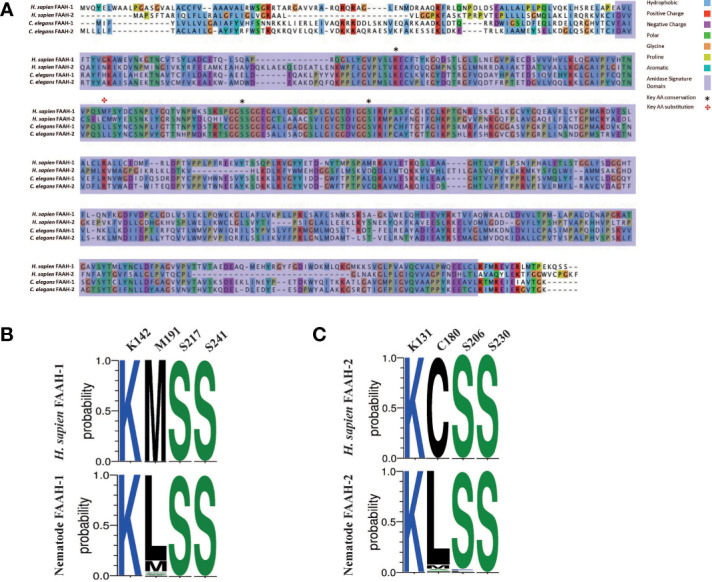
Nematode FAAH homologs conserve key functional domains and motifs, but display conservative substitutions in a key AEA binding site. **(A)** Protein sequence alignment of *Homo sapiens* fatty acid amide hydrolase (FAAH-1), *H. sapiens* FAAH-2, *Caenorhabditis elegans* FAAH-1 and *C. elegans* FAAH-2. Amino acids are highlighted in the same colour if > 60% of residues are conserved. Legend denotes conserved amino residues, key ligand binding residues in FAAH-1 and FAAH-2, and the amidase signature domain. AA denotes amino acid; *H. sapiens* denotes *Homo sapiens*, *C. elegans* denotes *Caenorhabditis elegans*. **(B)** Amino acid sequence-logo representing sequence diversity between key residues in nematode FAAH-1 homologs (consensus) vs *H. sapiens* FAAH-1 [O00519, FAAH1_HUMAN] and, **(C)** Amino acid sequence-logo representing sequence diversity between key residues in nematode FAAH-2 homologs vs *H. sapiens* FAAH-2. Key EC binding site residues were derived from *H. sapiens* FAAH-1 and -2 and are detailed in the top column of each table. Colours indicate hydrophobicity of amino acid residues (hydrophilic residues are blue, neutral residues are green and hydrophobic residues are black).

The mammalian AEA hydrolysis enzymes FAAH-1 and FAAH-2 possess four key residues required for catabolic activity (FAAH-1: K142, M191, S217, S241; FAAH-2: K131, C180, S206, S230) (42, 91, 92). Analysis of nematode FAAH-1 homologs identified in this study (see **Figure 2**) revealed that >90% possess the key mammalian binding site residues K142, S217 and S241, while 83% of identified nematode FAAH-2 homologs possess K131, S206 and S230 (see **Figures 4A–C**). However, 87% of nematode FAAH-1 homologs display a conservative substitution (methionine for leucine) at position 191 while 80% of nematode FAAH-2 homologs substitute cysteine for leucine at position 180 (**Figures 4A–C**). M191 has been implicated in mammalian FAAH-1 EC-derivative binding, with studies demonstrating lipophilic interactions between this residue and partial cannabinoid receptor agonists, linking it to AEA binding (93), however the significance of C180 (FAAH-2) is less clear (94–96).

Mammalian FAAH-1 and -2 also possess a highly conserved 130 bp amidase signature domain that enables enzyme characterisation (97, 98); this domain is conserved in 94% of the nematode species examined in this study (**Figure 4A**).

While these data demonstrate that nematodes possess homologs for FAAH enzymes that display broad conservation with vertebrates, the presence of a distinct substitution in a key AEA binding site across many nematodes may highlight the potential for drug target selectivity towards parasitic nematode species.

### Nematodes Possess Homologs for the Mammalian 2-AG Degradation Enzyme ABHD-12

In mammals, MAGL is primarily responsible for 2-AG degradation and thus the termination of EC signalling (31, 99). Additional 2-AG degradation enzymes, ABHD-6 and -12, have also been reported (33, 100) however, ABHD-6 is thought to play a less significant role in 2-AG degradation (31, 99).

The nematode literature presents conflicting data on the identity of the 2-AG degradation enzyme; indeed prior to this study it was unclear whether nematodes possess a true ortholog of MAGL (19, 27) or, if the nematode 2-AG degradation enzyme is actually an ABHD-12 ortholog (11, 18). It is interesting to note that previous work indicates that *magl* homologs are absent in some nematode species (18), which has been confirmed here using our pan-phylum *in silico* approach (**Figure 2A**). Indeed, in our analyses only two nematode species (of the 109 in this study) returned an *magl*-like sequence within the top 5 BLASTp hits (*Strongylus vulgaris*, SVUK_0001964001; *Steinernema scapterisci*, L892_g30127.t1), both of which failed to meet E-value inclusion criteria. The absence of MAGL in nematodes indicates the presence of an alternative 2-AG degradation enzyme.

**Figure 5:**
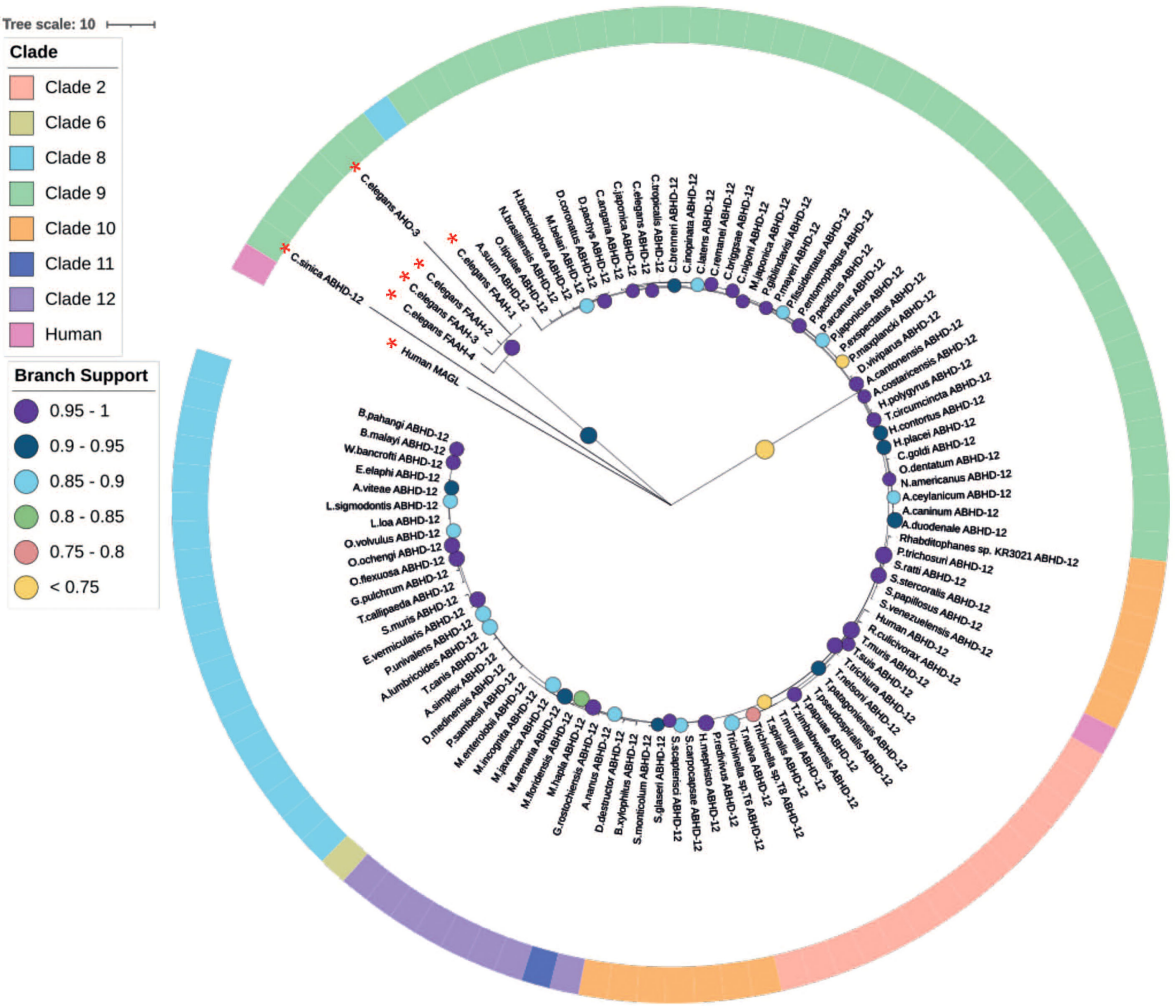
Maximum likelihood phylogeny of nematode ABHD-12 homologs. 98 nematode lysophosphatidylserine lipase alpha/beta-hydrolase domain containing-12 (ABHD-12) homologs are shown in addition to *Homo sapiens* ABHD-12 [Q8N2K0 (ABD12_HUMAN)] *H. sapiens* monoacylglycerol lipase (MAGL) [Q99685 (MGLL_HUMAN)], *Caenorhabditis elegans* fatty acid amide hydrolase (FAAH-1-4) [WBGene00015047, WBGene00015048, WBGene00019068, WBGene00013232] and *C. elegans* AHO-3 [WBGene00045192; alpha/beta hydrolase containing protein]. Non-ABHD-12 homologs are marked with a red asterisk (*). Outer ring denotes nematode clade and coloured circles represent branch support values. Tree was generated from an alignment trimmed to include protein functional domains. Branch supports indicate statistical support from approximate likelihood ratio test (aLRT).

In light of the limited role for ABHD-6 in mammalian 2-AG degradation and the absence of MAGL across nematodes (as reported here and in previous studies), we focused our attention on ABHD-12 as a putative alternative to MAGL in nematodes. Pan-phylum analysis of nematode genomes identified orthologs for ABHD-12 in 88% of nematode species examined. Significantly, phylogenetic analyses of these putative *abhd-12* homologs demonstrated that 99% of nematode BLAST returns for *abhd-12* cluster more strongly with human *abdh-12* than human *magl* (**Figure 5** and **Supplementary Figure 1**) suggesting that the nematode 2-AG hydroxylase enzymes identified here, and originally designated as *magl* in *C. elegans* [Y97E10AL.2; (19)], are orthologous with *abhd-12*.

### Nematode NPR-19 and -32 Orthologs Possess Key Functional Motifs and EC Binding Residues

NPR-19 has been identified as a putative EC-GPCR in *C. elegans* and appears to modulate key aspects of nematode biology including axon regeneration, locomotion, modulation of noiceception and feeding, and therefore may represent a promising anthelmintic target (27).

**Figure 6:**
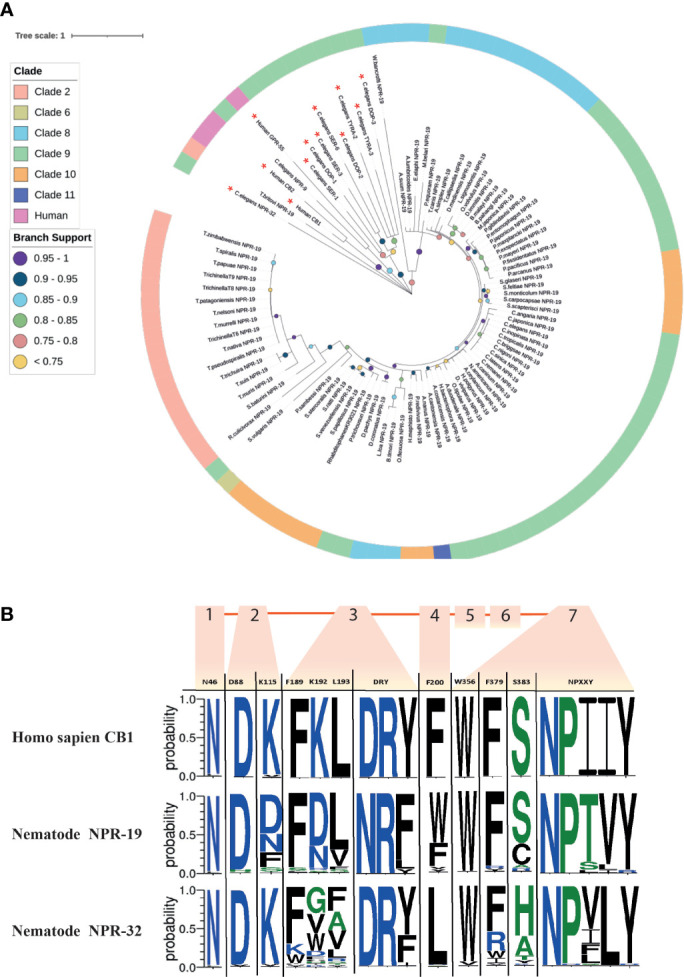
Maximum likelihood phylogeny of nematode *npr-19* homologs. **(A)** 85 nematode NPR*-*19 homologs are shown in addition to *Homo sapiens* CB1 and CB2 [P21554 (CNR1_HUMAN), P34972 (CNR2_HUMAN)], *H. sapiens* GPR-55 [Q9Y2T6 (GPR55_HUMAN)] and several *Caenorhabditis elegans* biogenic amine receptors (serotonin [SER-1,-3,-6; WBGene00004776, WBGene00004778, WBGene00021897] dopamine [DOP-1-3; WBGene00001052, WBGene00001053, WBGene00020506] and tyramine [TYRA-2 and -3; WBGene00017157, WBGene00006475]). Non-NPR*-*19 homologs are marked with a red asterisk (*). Outer ring denotes nematode clade and coloured circles represent branch support values. Tree was generated from an alignment trimmed to include functional domains. Branch supports indicate statistical support from approximate likelihood ratio test (aLRT). **(B)** Amino acid sequence-logo demonstrating sequence diversity between nematode NPR-19 and NPR-32 orthologs (consensus) and *H. sapiens* CB1 [P21554 (CNR1_HUMAN)]. Known vertebrate endocannabinoid binding and GPCR motifs/residues are indicated in the top row of the sequence logo table, transmembrane regions 1-7 are indicated by orange boxes and numbers, amino acid colours indicate hydrophobicity of amino acid residues (hydrophilic residues are blue, neutral residues are green and hydrophobic residues are black).

78% of the nematodes examined in this study possess an *npr-19* ortholog (see **Figure 2** and **Figure 6A**). Phylogenetic analysis demonstrates that nematode *npr-19* orthologs failed to cluster with human CB1 and CB2, confirming that NPR-19 is not the direct ortholog of the human EC-GPCRs receptors (CB1 and CB2) (**Figure 6A** and **Figure SI 1**). Further analysis of the nematode *npr-19* orthologs revealed that whilst the nematode NPR-19 consensus sequence has only 23% similarity to human CB1, several of the known human CB1 EC binding residues (N46, D88, F189, L193, F379, S383) (101, 102) are conserved (**Figure 6B**). This indicates that nematode NPR-19 orthologs are likely to possess EC ligand binding capacity and aligns with previous work in *C. elegans* (27). When examined at the clade level, sequence analysis showed that two conservative substitutions are present at position K192 (82% of clade 2 species substitute K192 for N192), while species in clades 8, 9, 10 and 12 substitute K192 for D192. K192 forms a hydrogen bond with the amide oxygen of AEA, implicating this residue in ligand binding (101) (see **Figure 6B**).

NPR-32 is implicated in *C. elegans* axon regeneration (17) and, in addition to NPR-19, is believed to be a putative EC-GPCR (27). In this study we identified 97 NPR-32 homologs (**Figure 2** and **Supplementary Figure 1**) which share (consensus sequence) only 21% identity with the human EC-GPCR CB1, but conserve several key residues (N46, D88, K115, F189; **Figure 6B**) that are believed to be important for ECS function (17).

In addition, CB1 possesses a “toggle switch” (residue W356), a putative molecular hinge that interacts with F200 to change the form and state of the receptor which in turn aids EC ligand binding (103, 104). In nematodes the “toggle switch” W356 is conserved in >85% of NPR-19 and -32 orthologs, whereas NPR-19 F200 is substituted for W200 in 59% of species and NPR-32 F200 is substituted for L200 in 90% of species, (see **Figure 6B**). The significance of these observations will be revealed through molecular docking studies, crystal structure analysis, and functional genomics in relevant parasite species, and will inform the role and importance of these receptors in nematode ECS biology.

### EC-effectors Are Differentially Expressed Across Nematode Life Stages, Sexes and Tissues, Suggesting Key Roles in Parasite Biology

EC-effector expression has previously been examined in several parasites demonstrating differential expression across life stages (18). Here we further profiled EC-effector expression in 32 nematode species representing several distinct lifestyles (see **File SI 2**).

Our data demonstrate that several of the putative ECRs examined in this study are upregulated in third-stage larvae (L3) of several parasites including *Ancylostoma ceylanicum, Teladorsagia circumcincta, Dictyocaulus viviparus, H. contortus, S. ratti, S. stercoralis* and *Onchocerca volvulus* (see **Supplementary Figure 2**). Parasitic nematode L3 larvae are analogous to the dauer life stage of *C. elegans*; both *C. elegans* dauer and parasitic nematode L3 stages display similar physiology, are in arrested development, are non-feeding and are highly resistant to their environment (105–107). L3 parasites of species such as those outlined above transition from arrested (dauer-like) L3 larvae to infective L3 (iL3) larvae either constitutively or *via* the influence of host and environmental factors (108). The ECS pathway has been implicated in antagonization of dauer formation and abolishing dauer larval arrest *via* stimulation of cholesterol in *C. elegans* and, in turn, promotion of nematode growth and development (15). Thus, the upregulation of putative ECRs in L3 stages of parasitic nematodes could suggest an analogous role for EC signalling in parasite growth and development at a critical stage in the parasitic lifecycle.

Upregulation of putative ECRs, and a key ECE (*dagl-2*) associated with EC ligand biosynthesis, is evident in *Strongyloides* iL3s (see **Supplementary Figure 2**). In contrast, the ECEs responsible for EC ligand degradation (*abhd-12* and *faah-1-4*) are downregulated at the iL3 stage (see **Supplementary Figure 2**). The opposite expression profile is noted in the adult life stage (free-living and parasitic females) of *Strongyloides* spp. where EC-degradation enzymes are upregulated and putative ECRs and *dagl-2* are downregulated (see **Supplementary Figure 2**). These data suggest that higher levels of EC ligands may exist in the iL3 stage of *S. ratti* and *S. stercoralis* and is consistent with the elevated production of EC-ligands by *N. brasiliensis* iL3s (18). Together these data indicate that the ECS system may be involved in processes linked to host infection. Parasitic nematodes exploit numerous sensory cues and mechanisms in order to find their host (109), thus the upregulation of EC-effectors in iL3s may also implicate EC signalling in sensory perception, host-seeking, and the establishment of host infection. These data will direct future functional genomics studies around the role of EC signalling in host finding and infection in tractable parasitic nematodes.

Expression profiling of EC-effectors in sex-specific transcriptome data reveal differential expression patterns in male and female nematodes of several species (see **Supplementary Figure 2**). In *T. circumcincta* EC-ligand degradation enzymes are broadly downregulated in adult males, and upregulated in adult females (**Supplementary Figure 2**). Conversely, *O. volvulus* exhibits upregulation of all pathway components in adult males, and downregulation in adult females (**Supplementary Figure 2**). Sex-specific expression of EC-effectors is common in mammalian species, where they exhibit alternative actions on neuropsychiatric processes and reproductive events (110–112). In addition the ECS pathway has been implicated in mammalian fertility regulation (113–117) and in invertebrate reproduction (118, 119). Interrogation of expression at the tissue level is challenging in nematodes where data sets are limited to species which are readily amenable to dissection, for example *Ascaris suum* and *Dirofilaria immitis*. Whilst it would appear that EC-effectors are upregulated in reproductive tissues (e.g. in *A. suum*), further analysis across more tissue types and species is required before meaningful comparisons can be made (data not shown). While the role of EC signalling in the regulation of vertebrate and invertebrate reproduction has been documented, ECS system function in nematode reproduction is yet to be determined. Indeed, enhancing the ability to generate life- and tissue-specific data from key parasitic nematodes will inform functional biology.

## Conclusions

*In silico* approaches and the proliferation of nematode omics resources provide a valuable opportunity to identify putative novel anthelmintic drug targets for the control of parasite disease. This study focuses on the characterisation of the nematode ECS pathway, driven by its putative biological importance and therapeutic appeal (16, 27, 28, 39, 42). Here we: (i) provide a comprehensive pan-phylum overview of EC-effector complements in nematodes, that represent divergent clades and lifestyles; (ii) unravel the complexity of the nematode ECS to identify putative species- and lifestyle-specific EC-pathways and drug target selectivity, and (iii) reveal life stage-, and sex-specific EC-effector expression patterns in relevant parasite species. These data will direct the selection of novel ECS pathway targets for functional validation efforts in parasitic nematodes to inform biology and anthelmintic drug discovery pipelines.

## Data Availability Statement

The datasets presented in this study can be found in online repositories. The names of the repository/repositories and accession number(s) can be found in the article/**Supplementary Material**.

## Author Contributions

LA, AM, AGM, and NM designed the research. BC, DM and LC, performed the research. BC, DM and LC, analysed the data with assistance from CM and PM. LA, AM, BC, AGM and NM wrote the manuscript. All authors contributed to the article and approved the submitted version.

## Funding

This work was supported by: the Academy of Medical Sciences Springboard Award (SBF004\1018 to LA); the Biotechnology and Biological Sciences Research Council (BB/H019472/1 to AM); the Biotechnology and Biological Sciences Research Council/Boehringer Ingelheim (BB/T016396/1 to AM, NM, AGM, and LA); the Department of Education and Learning for Northern Ireland (studentships awarded to BC and LC); the Department of Agriculture, Environment and Rural Affairs for Northern Ireland (studentship awarded to DM).

## Conflict of Interest

The authors declare that the research was conducted in the absence of any commercial or financial relationships that could be construed as a potential conflict of interest.

## Publisher’s Note

All claims expressed in this article are solely those of the authors and do not necessarily represent those of their affiliated organizations, or those of the publisher, the editors and the reviewers. Any product that may be evaluated in this article, or claim that may be made by its manufacturer, is not guaranteed or endorsed by the publisher.

## Supplementary Material

File SI 1
*Caenorhabditis* spp. EC-effector gene IDs. List of EC-effector gene IDs from *Caenorhabditis* spp. that were used as query sequences in this study.

File SI 2 Nematode genome and transcriptome accession numbers and citations. List of accession numbers for all genome and transcriptome datasets used in this study along with original citations.

File SI 3 Nematode HMM/BLAST hit gene IDs. List of all BLAST hit gene IDs generated in this study.

File SI 4 Presence of alternative AEA synthesis pathways in NAPE encoding species. Presence/absence list of alternative AEA synthesis pathway enzymes in 20 key species that do possess NAPE orthologs (Tab 1) and BLAST hit gene IDs (Tab 2).

Supplementary Table 1 Table of EC-effectors included in study.

Supplementary Table 2 Table of EC-effector motifs and relevant citations.

Supplementary Figure 1 Maximum likelihood phylogeny of: **(A)** 98 nematode ABHD-12 homologs. *Homo sapiens* ABHD-12 *** *** [Q8N2K0 (ABD12_HUMAN)] *H. sapiens* MAGL* *[Q99685(MGLL_HUMAN)],* Caenorhabditis elegans *FAAH-*1-4* [WBGene00015047, WBGene00015048, WBGene00019068, WBGene00013232] and *C. elegans *AHO-3 [WBGene00045192; alpha/beta hydrolase containing protein] also included; **(B)** 85 nematode NPR-19 homologs. *Homo sapiens* CB1 and CB2 [P21554 (CNR1_HUMAN), P34972 (CNR2_HUMAN)], *H. sapiens* GPR-55* *[Q9Y2T6 (GPR55_HUMAN)] and several *C. elegans *biogenic amine receptors (serotonin [SER-1,-3,-6; WBGene00004776, WBGene00004778, WBGene00021897] dopamine [DOP-1-3; WBGene00001052, WBGene00001053, WBGene00020506] and tyramine [TYRA-2 and -3; WBGene00017157, WBGene00006475]) also included. **(C)** 89 nematode NPR-9 homologs. *Homo sapiens* CB1 and CB2 [P21554 (CNR1_HUMAN), P34972 (CNR2_HUMAN)], *H. sapiens* GPR-55 [Q9Y2T6 (GPR55_HUMAN)] and several *C. elegans* biogenic amine receptors (serotonin [SER-1,-3,-6; WBGene00004776, WBGene00004778, WBGene00021897] dopamine [DOP-1-3; WBGene00001052, WBGene00001053, WBGene00020506] and tyramine [TYRA-2 and -3; WBGene00017157, WBGene00006475]) also included. **(D)** 97 nematode NPR-32 homologs. *Homo sapiens* CB1 and CB2 [P21554 (CNR1_HUMAN), P34972 (CNR2_HUMAN)], *H. sapiens* GPR-55 [Q9Y2T6 (GPR55_HUMAN)] and several *C. elegans* biogenic amine receptors (serotonin [SER-1,-3,-6; WBGene00004776, WBGene00004778, WBGene00021897] dopamine [DOP-1-3; WBGene00001052, WBGene00001053, WBGene00020506] and tyramine [TYRA-2 and -3; WBGene00017157, WBGene00006475]) also included. **(E)** 78 nematode NHR-49 homologs. *Homo sapiens* PPARG [P37231 (PPARG_HUMAN)], *H. sapiens* PPARD [Q03181 (PPARD_HUMAN)], *H. sapiens* PPARA [Q07869 (PPARA_HUMAN)], *C. elegans* NHR-88 [WBGene00003678], *C. elegans* NHR-64 [WBGene00003654] and *C. elegans* NHR-35 [WBGene00003628] also included. **(F)** 107 nematode OCR-2 homologs. *Homo sapiens* TRPV1-3 [Q8NER1 (TRPV1_HUMAN), Q9Y5S1 (TRPV2_HUMAN), Q8NET8 (TRPV3_HUMAN)], alongside *C. elegans* OSM-9 [WBGene00003889], *C. elegans* OCR-1 [WBGene00003838], *C. elegans* OCR-3 [WBGene00003840] and *C. elegans* UNC-44 [WBGene00006780] also shown. **(G)** 99 nematode OCTR-1 homologs. *Homo sapiens* CB1 and CB2 [P21554 (CNR1_HUMAN), P34972 (CNR2_HUMAN)], *H. sapiens* GPR-55 [Q9Y2T6 (GPR55_HUMAN)], *H. sapiens* ADA2A-C [P08913 (ADA2A_HUMAN). P18089 (ADA2B_HUMAN), P18825 (ADA2C_HUMAN)] and several *C. elegans* biogenic amine receptors (serotonin [SER-1,-3,-6; WBGene00004776, WBGene00004778, WBGene00021897] dopamine [DOP-1-3; WBGene00001052, WBGene00001053, WBGene00020506] and tyramine [TYRA-2 and -3; WBGene00017157, WBGene00006475]) also shown. **(H)** 100 nematode SER-4 homologs. *Homo sapiens* CB1 and CB2 [P21554 (CNR1_HUMAN), P34972 (CNR2_HUMAN)], *H. sapiens* GPR-55 [Q9Y2T6 (GPR55_HUMAN) and several *C. elegans* biogenic amine receptors (serotonin [SER-1,-3,-6; WBGene00004776, WBGene00004778, WBGene00021897] dopamine [DOP-1-3; WBGene00001052, WBGene00001053, WBGene00020506] and tyramine [TYRA-2 and -3; WBGene00017157, WBGene00006475]) also shown. Outer colours represent nematode clade and circles represent branch support values. Tree generated from an alignment trimmed to include functional domains. Branch supports indicate statistical support from approximate likelihood ratio test (aLRT).

Supplementary Figure 2 Life stage and sex-specific expression profiles of EC-effectors. **(A)** Ancylostoma ceylanicum, **(B)** Teladorsagia circumcincta, **(C)** Dictyocaulus viviparus, **(D)** Haemonchus contortus, **(E)** Strongyloides ratti, **(F)** Strongyloides stercoralis, **(G)** Trichuris muris and **(H)** Onchocerca volvulus. Expression heatmaps generated from log2TPM values of all EC-effector transcripts. Average Clustering Method & Pearson’s Distance Measurement Method employed. Life stage and sex-specific data are arranged in columns, rows indicate individual EC-effector transcripts as denoted by effector abbreviation/gene ID. Coloured circles represent EC-receptors (orange), EC biosynthesis enzymes (green) and EC degradation enzymes (purple). [Life stages include; L1, L2, activated L3 (L3A), not activated L3 (L3NA), untreated L3 (L3UT), adult female (AF), adult male (AM) hypobiotic larvae (Lhyp), mixed L1 & L2 (L1+L2), pre-adult L5 female (L5AF), pre-adult L5 male (L5AM), pre-adult L5 mixed gender (L5 Mixed), Adult L5 (L5A), infective larvae (iL3), free-living female (FL Female), tissue migrating L3 (L3+), parasitic female (P Female), post-free living L1 (PFLL1), post-parasitic L1 (PPL1), post-parasitic L3 (PPL3)].

## Abbreviations

2-AG, 2-arachidonoylglycerol; ABHD-4, Abhydrolase Domain Containing 4, N-Acyl Phospholipase B; ABHD-5, Abhydrolase Domain Containing 5; ABHD-6, Abhydrolase Domain Containing 6; ABHD-12, Abhydrolase Domain Containing 12, Lysophospholipase; AEA, Anandamide/N-arachidonoylethanolamine; BUSCO, Benchmarking Universal Single-Copy Orthologs; CB1, Cannabinoid Receptor 1; CB2, Cannabinoid Receptor 2; CEGMA, Core Eukaryotic Genes Mapping Approach; DAGL, Diacylglycerol lipase; EC, Endocannabinoid; EC-GPCR, Endocannabinoid G-protein coupled receptor; ECEs, Endocannabinoid Enzymes; ECRs, Endocannabinoid Receptors; ECS, Endocannabinoid signalling; FAAH-1, Fatty Acid Amide Hydrolase 1; FAAH-2, Fatty Acid Amide Hydrolase 2; GDE-1,Glycerophosphodiester phosphodiesterase 1; Glycero-p-AEA, Glycerophosphoanandamide; GPR-55, G-protein coupled receptor 55; HMM,Hidden Markov Model; Lyso-NAPE, N-acyl-1-acyl-lyso-PE; Lyso-PLD, Lysophospholipase D; MAGL, Monoacylglycerol-lipase; MSA, Multiple Sequence Alignment; NAPE, N-arachidonyl phosphatidyl ethanol-phospholipase D; NAPE-1, N-acyl phosphatidylethanolamine-specific phospholipase-1; NAPE-2, N-acyl phosphatidylethanolamine-specific phospholipase-2; NAPE-PLD, N-Acyl Phosphatidyl Ethanolamine specific phospholipase D; NHR-49, Nuclear Hormone Receptor-49; NPR-9, Neuropeptide Receptor-9; NPR-19, Neuropeptide Receptor-19; NPR-32, Neuropeptide Receptor-32; OCR-2, Osin-9 and Capsacin receptor-related 2; OCTR-1, Octopamine Receptor-1; PLA-2, Phospholipase A2; SER-4, Serotonin/Octopamine receptor family-4; TRPV, Transient receptor potential vanilloid channel.
