# Supplementary material for: Pan-phylum In Silico Analyses of Nematode Endocannabinoid Signalling Systems Highlight Novel Opportunities for Parasite Drug Target Discovery
Source: Front Endocrinol (Lausanne). 2022 Jul 1;13:892758. doi: 10.3389/fendo.2022.892758 (PMC9283691; doi:10.3389/fendo.2022.892758)
Supplement: File SI 1 — Caenorhabditis spp. EC-effector gene IDs. List of EC-effector gene IDs from Caenorhabditis spp. that were used as query sequences in this study. [file DataSheet_1.zip › Table SI 1.DOCX]

| **EC Effector in *C. elegans* (Proposed Human Ortholog)** | **Proposed Role in nematodes (predominantly derived from *C. elegans*)** | **Citations** |
| --- | --- | --- |
| NPR-9 [G-protein Coupled Receptor 55 (GPR55)] | - Initiation and fine-tuning of backwards locomotion and reversals - Negative regulation of the immune response - Roles in roaming and foraging behaviours | (Bendena et al., 2008, Campbell et al., 2016, Yu et al., 2018) |
| NPR-19 [Cannabinoid Receptor 1 (CB1), GPR55] | - Roles in nociception, locomotion and feeding - Inhibition of axon regeneration | (Pastuhov et al., 2016, Oakes et al., 2017) |
| NPR-32 (CB1) | - Inhibition of axon regeneration | (Pastuhov et al., 2016) |
| NHR-49 [Peroxisome proliferator-activated receptor gamma (PPARY)] | - Control of consumption and balance of fat - Oxidation and desaturation of fatty acids | (Atherton et al., 2008) |
| OCR-2 [Transient Receptor Potential Cation Channel Subfamily V Member 1 [TRPV1)] | - Implicated in larval starvation survival and adult lifespan - Chemosensory functions - Inhibition of aversive behaviour | (Jose et al., 2007, Lee and Ashrafi, 2008) |
| SER-4 [5-Hydroxytryptamine 1A (HTR1A)] | - Roles in nociception and locomotion | (Oakes et al., 2017) |
| OCTR-1 [Alpha-2A adrenergic receptor (ADRA2A)] | - Regulation of innate immunity via suppression of translation - Nociception and locomotion | (Liu et al., 2016, Oakes et al., 2017) |
| NAPE-1/NAPE-2 (N-acyl-phosphatidylethanolamine-hydrolysing phospholipase D) | - NAE biosynthesis - Over-expression has a temperature dependent effect on development/life-span | (Harrison et al., 2014) |
| FAAH-1 (Fatty acid amide hydrolase 1) | - NAE degradation - Over-expression resulted in developmental delays | (Harrison et al., 2014) |
| FAAH-2 (Fatty acid amide hydrolase 2) | - NAE degradation | (Harrison et al., 2014) |
| DAGL-2 (Diacylglycerol lipase 2) | - Synthesis of 2-AG - Over-expression extends mean lifespan by up to 13% | (Lin et al., 2014) |
| ABHD-12 (Monoacylglycerol lipase 2) | - Degradation of 2-AG | (Savinainen et al., 2012, Wei et al., 2016) |

**REFERENCES**

Atherton, H. J., Jones, O. A., Malik, S., Miska, E. A. & Griffin, J. L. 2008. A comparative metabolomic study of NHR-49 in Caenorhabditis elegans and PPAR-α in the mouse. *FEBS letters,* 582**,** 1661-1666.

Bendena, W. G., Boudreau, J. R., Papanicolaou, T., Maltby, M., Tobe, S. S. & Chin-Sang, I. D. 2008. A Caenorhabditis elegans allatostatin/galanin-like receptor NPR-9 inhibits local search behavior in response to feeding cues. *Proceedings of the National Academy of Sciences,* 105**,** 1339-1342.

Campbell, J. C., Polan-Couillard, L. F., Chin-Sang, I. D. & Bendena, W. G. 2016. NPR-9, a galanin-like G-protein coupled receptor, and GLR-1 regulate interneuronal circuitry underlying multisensory integration of environmental cues in Caenorhabditis elegans. *PLoS genetics,* 12.

Harrison, N., Lone, M. A., Kaul, T. K., Rodrigues, P. R., Ogungbe, I. V. & Gill, M. S. 2014. Characterization of N-acyl phosphatidylethanolamine-specific phospholipase-D isoforms in the nematode Caenorhabditis elegans. *PloS one,* 9.

Jose, A. M., Bany, I. A., Chase, D. L. & Koelle, M. R. 2007. A specific subset of transient receptor potential vanilloid-type channel subunits in Caenorhabditis elegans endocrine cells function as mixed heteromers to promote neurotransmitter release. *Genetics,* 175**,** 93-105.

Lee, B. H. & Ashrafi, K. 2008. A TRPV channel modulates C. elegans neurosecretion, larval starvation survival, and adult lifespan. *PLoS genetics,* 4.

Lin, Y. H., Chen, Y. C., Kao, T. Y., Lin, Y. C., Hsu, T. E., Wu, Y. C., Ja, W. W., Brummel, T. J., Kapahi, P. & Yuh, C. H. 2014. Diacylglycerol lipase regulates lifespan and oxidative stress response by inversely modulating TOR signaling in D rosophila and C. elegans. *Aging Cell,* 13**,** 755-764.

Liu, Y., Sellegounder, D. & Sun, J. 2016. Neuronal GPCR OCTR-1 regulates innate immunity by controlling protein synthesis in Caenorhabditis elegans. *Scientific reports,* 6**,** 36832.

Matúš, D. & Prömel, S. 2018. G Proteins and GPCRs in C. elegans Development: A Story of Mutual Infidelity. *Journal of developmental biology,* 6**,** 28.

Oakes, M. D., Law, W. J., Clark, T., Bamber, B. A. & Komuniecki, R. 2017. Cannabinoids activate monoaminergic signaling to modulate key C. elegans behaviors. *Journal of Neuroscience,* 37**,** 2859-2869.

Pastuhov, S. I., Hisamoto, N. & Matsumoto, K. 2015. MAP kinase cascades regulating axon regeneration in C. elegans. *Proceedings of the Japan Academy, Series B,* 91**,** 63-75.

Pastuhov, S. I., Matsumoto, K. & Hisamoto, N. 2016. Endocannabinoid signaling regulates regenerative axon navigation in Caenorhabditis elegans via the GPCRs NPR‐19 and NPR‐32. *Genes to Cells,* 21**,** 696-705.

Sassa, T., Murayama, T. & Maruyama, I. N. 2013. Strongly alkaline pH avoidance mediated by ASH sensory neurons in C. elegans. *Neuroscience letters,* 555**,** 248-252.

Savinainen, J., Saario, S. & Laitinen, J. 2012. The serine hydrolases MAGL, ABHD6 and ABHD12 as guardians of 2‐arachidonoylglycerol signalling through cannabinoid receptors. *Acta physiologica,* 204**,** 267-276.

Wei, M., Zhang, J., Jia, M., Yang, C., Pan, Y., Li, S., Luo, Y., Zheng, J., Ji, J. & Chen, J. 2016. α/β-Hydrolase domain-containing 6 (ABHD6) negatively regulates the surface delivery and synaptic function of AMPA receptors. *Proceedings of the National Academy of Sciences,* 113**,** E2695-E2704.

Yu, Y., Zhi, L., Wu, Q., Jing, L. & Wang, D. 2018. NPR-9 regulates the innate immune response in Caenorhabditis elegans by antagonizing the activity of AIB interneurons. *Cellular & molecular immunology,* 15**,** 27-37.
