# Supplementary material for: Pan-phylum In Silico Analyses of Nematode Endocannabinoid Signalling Systems Highlight Novel Opportunities for Parasite Drug Target Discovery
Source: Front Endocrinol (Lausanne). 2022 Jul 1;13:892758. doi: 10.3389/fendo.2022.892758 (PMC9283691; doi:10.3389/fendo.2022.892758)
Supplement: File SI 1 — Caenorhabditis spp. EC-effector gene IDs. List of EC-effector gene IDs from Caenorhabditis spp. that were used as query sequences in this study. [file DataSheet_1.zip › Table SI 2.DOCX]

| **EC-effector in *C. elegans* (Proposed Human Ortholog)** | **Functional motif(s)** | **Key amino acid residues** | **Proposed role(s) of motifs** | **Proposed role(s) of key amino acid residues** | **Citations** |
| --- | --- | --- | --- | --- | --- |
| NPR-9 (G-protein Coupled Receptor 55 [GPR55]) | DRY  CWXP  S(N)LAXXAD  NPXXY | Y101, P241, F246 | - DRY – highly conserved motif on transmembrane helix (TMH) 3, role in receptor activation and stabilisation of active states and influence on signal transduction - CWXP – putative molecular hinge essential for recognition of ligands - S(N)LAXXAD – roles in stabilisation of interhelical interaction between TMH2-TMH3/TMH4 - NPXXY- critical for receptor activation | - Y101, P241, F246 – conserved amino acids between GPR-55 and NPR-9; linked directly to AEA binding, Y101 acts as a toggle switch | (Ballesteros and Weinstein, 1995, Pei et al., 2008, Shim, 2009, Shim et al., 2011, Stadel et al., 2011, Lingerfelt et al., 2017, Zhang et al., 2018, Kumar et al., 2019) |
| NPR-19 (Cannabinoid Receptor 1 [CB1], GPR55) | DRY  CWXP  S(N)LAXXAD  NPXXY | F189, K192, L193, F379, S383 | - DRY – highly conserved motif on transmembrane helix (TMH) 3, role in receptor activation and stabilisation of active states and influence on signal transduction - CWXP – putative molecular hinge essential for recognition of ligands - S(N)LAXXAD – roles in stabilisation of interhelical interaction between TMH2-TMH3/TMH4 - NPXXY- critical for receptor activation | - F189 – interacts with AEA amide oxygen; mutation in CB1 decreases AEA binding and affinity sixfold - K192 – interacts with AEA amide oxygen - S383 – forms a hydrogen bond with AEA hydroxyl - F189, L193, F379, S383 – included in AEA binding pocket | (Ballesteros and Weinstein, 1995, McAllister et al., 2003, McAllister et al., 2004, Pei et al., 2008, Shim, 2009, Reggio, 2010, Shim et al., 2011, Stadel et al., 2011, Oakes et al., 2017, Zhang et al., 2018) |
| NPR-32 (CB1) | DRY  CWXP  S(N)LAXXAD  NPXXY | N46, D88, K115 | - DRY – highly conserved motif on transmembrane helix (TMH) 3, role in receptor activation and stabilisation of active states and influence on signal transduction - CWXP – putative molecular hinge essential for recognition of ligands - S(N)LAXXAD – roles in stabilisation of interhelical interaction between TMH2-TMH3/TMH4 - NPXXY- critical for receptor activation | - N46, D88, K115 – implicated in AEA binding | (Ballesteros and Weinstein, 1995, Pei et al., 2008, Shim, 2009, Shim et al., 2011, Stadel et al., 2011, Pastuhov et al., 2016, Zhang et al., 2018) |
| NHR-49 (Peroxisome proliferator-activated receptor gamma [PPARY]) | 9aaTAD motif [positions 495-503] | Q286, S289, H323, H449, Y473 | - 9aaTAD motif – activates transcription as a small peptide; conserved across nuclear hormone receptors | - Q286, S289, H323, H449, Y473 – ligands form hydrogen bonds with these residues in a hydrophilic pocket implicated in agonist binding | (Sheu et al., 2005, Atherton et al., 2008, Piskacek et al., 2019) |
| OCR-2 (Transient Receptor Potential Cation Channel Subfamily V Member 1 [TRPV1]) | CRAC motif [YYTR; positions 553-557] | R491, Y511, S512, T550, R557, E637, D647, E649 | - CRAC motif [YYTR] – conference of cholesterol sensitivity | - T550, Y511 – conference of vanilloid sensitivity and ligand binding - R491, Y511, S512 – molecular determinants and role in ligand binding - R557 – role in volted gate channel gating - E637, D647, E649 – alter receptor sensitivity to divalent cations | (Gavva et al., 2004, Jose et al., 2007, Fernandez-Ballester and Ferrer-Montiel, 2008, Picazo-Juarez et al., 2011) |
| SER-4 (5-Hydroxytryptamine 1A [HTR1A]) | DRY  CWXP  S(N)LAXXAD  NPXXY | I113, D116, V117, I189, F361, F362, Y390 | - DRY – highly conserved motif on transmembrane helix (TMH) 3, role in receptor activation and stabilisation of active states and influence on signal transduction - CWXP – putative molecular hinge essential for recognition of ligands - S(N)LAXXAD – roles in stabilisation of interhelical interaction between TMH2-TMH3/TMH4 - NPXXY- critical for receptor activation | - I113, V117, F361, F362 – hydrophobic interactions with ligands via nonpolar side chain - D116 – formation of salt bridge with ligand - I189 – forms hydrogen bonds with indole ring moiety - Y390 – forms hydrogen bond with centre of ligands | (Ballesteros and Weinstein, 1995, Pei et al., 2008, Shim, 2009, Stadel et al., 2011, Oakes et al., 2017, Zheng et al., 2017, Zhang et al., 2018) |
| OCTR-1 (Alpha-2A adrenergic receptor [ADRA2A]) | DRY  CWXP | D94, D128, D145, S214, S219, F423, F427 | - DRY – role in receptor activation and stabilisation of active states and influence on signal transduction - CWXP – putative molecular hinge essential for recognition of ligands | - D94, D128, D145, S214, S219, F427 – Role in ligand binding. Mutagenesis of these residues results in lower affinity for receptor agonists - F423 – in binding pocket | (Suryanarayana et al., 1991, Wang et al., 1991, Ballesteros and Weinstein, 1995, Pei et al., 2008, Oakes et al., 2017) |
| NAPE-1/NAPE-2 (N-acyl-phosphatidylethanolamine-hydrolysing phospholipase D) | HX(E/H)XD(C/R/S/H)X50–70HX15–30(C/S/D)X30–70H signature sequence | H253, D284, Q320, H321, H343 | - HX(E/H)XD(C/R/S/H)X50–70HX15–30(C/S/D)X30–70H signature sequence – role in zinc binding and hydrolysis | - H253, D284, Q320, H321, H343 – key involvement in catalytic processes | (Okamoto et al., 2004, Harrison et al., 2014) |
| FAAH-1 (Fatty acid amide hydrolase 1) | Amidase signature domain | K142, M191, S217, S241 | - Amidase signature domain – catalytic motif | - K142, S217, S241 – catalytic triad - M191 – inside active site | (Lucanic et al., 2011, Haq and Kilaru, 2020) |
| FAAH-2 (Fatty acid amide hydrolase 2) | Amidase signature domain | K131, C180, S206, S230 | - Amidase signature domain – catalytic motif | - K131, S206, S230 – catalytic triad - C180 – inside active site | (Lucanic et al., 2011, Sirrs et al., 2015, Haq and Kilaru, 2020) |
| DAGL-2 (Diacylglycerol lipase 2) | Serine lipase domain  PPXXF | S472, D524 | - Serine lipase domain – catalytic domain - PPXXF – consensus motif for binding coiled-coil domain of Homer proteins | - S472, D524 – inside active site | (Reisenberg et al., 2012) |
| ABHD-12 (Monoacylglycerol lipase 2) | Alpha/beta hydrolase  Domain [consists of  lipase motif (GTSMG)] | S122,S148, D230, D278, H269, H306 | - Lipase motif (GTSMG) – catalytic domain | - S122, D230, H269 – active site identified from mutagenesis studies - S148, D278, H306 – catalytic triad | (Karlsson et al., 1997, Savinainen et al., 2012) |

**REFERENCES**

Atherton, H. J., Jones, O. A., Malik, S., Miska, E. A. & Griffin, J. L. 2008. A comparative metabolomic study of NHR-49 in Caenorhabditis elegans and PPAR-α in the mouse. *FEBS letters,* 582**,** 1661-1666.

Ballesteros, J. A. & Weinstein, H. 1995. [19] Integrated methods for the construction of three-dimensional models and computational probing of structure-function relations in G protein-coupled receptors. *Methods in neurosciences.* Elsevier.

Fernandez-Ballester, G. & Ferrer-Montiel, A. 2008. Molecular modeling of the full-length human TRPV1 channel in closed and desensitized states. *Journal of Membrane Biology,* 223**,** 161-172.

Gavva, N. R., Klionsky, L., Qu, Y., Shi, L., Tamir, R., Edenson, S., Zhang, T., Viswanadhan, V. N., Toth, A. & Pearce, L. V. 2004. Molecular determinants of vanilloid sensitivity in TRPV1. *Journal of Biological Chemistry,* 279**,** 20283-20295.

Haq, I. & Kilaru, A. 2020. An endocannabinoid catabolic enzyme FAAH and its paralogs in an early land plant reveal evolutionary and functional relationship with eukaryotic orthologs. *Scientific reports,* 10**,** 1-14.

Harrison, N., Lone, M. A., Kaul, T. K., Rodrigues, P. R., Ogungbe, I. V. & Gill, M. S. 2014. Characterization of N-acyl phosphatidylethanolamine-specific phospholipase-D isoforms in the nematode Caenorhabditis elegans. *PloS one,* 9.

Jose, A. M., Bany, I. A., Chase, D. L. & Koelle, M. R. 2007. A specific subset of transient receptor potential vanilloid-type channel subunits in Caenorhabditis elegans endocrine cells function as mixed heteromers to promote neurotransmitter release. *Genetics,* 175**,** 93-105.

Karlsson, M., Contreras, J. A., Hellman, U., Tornqvist, H. & Holm, C. 1997. cDNA cloning, tissue distribution, and identification of the catalytic triad of monoglyceride lipase: evolutionary relationship to esterases, lysophospholipases, and haloperoxidases. *Journal of Biological Chemistry,* 272**,** 27218-27223.

Kumar, K. K., Shalev-Benami, M., Robertson, M. J., Hu, H., Banister, S. D., Hollingsworth, S. A., Latorraca, N. R., Kato, H. E., Hilger, D. & Maeda, S. 2019. Structure of a signaling cannabinoid receptor 1-G protein complex. *Cell,* 176**,** 448-458. e12.

Lingerfelt, M. A., Zhao, P., Sharir, H. P., Hurst, D. P., Reggio, P. H. & Abood, M. E. 2017. Identification of crucial amino acid residues involved in agonist signaling at the GPR55 receptor. *Biochemistry,* 56**,** 473-486.

Lucanic, M., Held, J. M., Vantipalli, M. C., Klang, I. M., Graham, J. B., Gibson, B. W., Lithgow, G. J. & Gill, M. S. 2011. N-acylethanolamine signalling mediates the effect of diet on lifespan in Caenorhabditis elegans. *Nature,* 473**,** 226.

Mcallister, S. D., Hurst, D. P., Barnett-Norris, J., Lynch, D., Reggio, P. H. & Abood, M. E. 2004. Structural mimicry in class AG protein-coupled receptor rotamer toggle switches: the importance of the F3. 36 (201)/W6. 48 (357) interaction in cannabinoid CB1 receptor activation. *Journal of Biological Chemistry,* 279**,** 48024-48037.

Mcallister, S. D., Rizvi, G., Anavi-Goffer, S., Hurst, D. P., Barnett-Norris, J., Lynch, D. L., Reggio, P. H. & Abood, M. E. 2003. An aromatic microdomain at the cannabinoid CB1 receptor constitutes an agonist/inverse agonist binding region. *Journal of medicinal chemistry,* 46**,** 5139-5152.

Oakes, M. D., Law, W. J., Clark, T., Bamber, B. A. & Komuniecki, R. 2017. Cannabinoids activate monoaminergic signaling to modulate key C. elegans behaviors. *Journal of Neuroscience,* 37**,** 2859-2869.

Okamoto, Y., Morishita, J., Tsuboi, K., Tonai, T. & Ueda, N. 2004. Molecular characterization of a phospholipase D generating anandamide and its congeners. *Journal of Biological Chemistry,* 279**,** 5298-5305.

Pastuhov, S. I., Matsumoto, K. & Hisamoto, N. 2016. Endocannabinoid signaling regulates regenerative axon navigation in Caenorhabditis elegans via the GPCRs NPR‐19 and NPR‐32. *Genes to Cells,* 21**,** 696-705.

Pei, Y., Mercier, R. W., Anday, J. K., Thakur, G. A., Zvonok, A. M., Hurst, D., Reggio, P. H., Janero, D. R. & Makriyannis, A. 2008. Ligand-binding architecture of human CB2 cannabinoid receptor: evidence for receptor subtype-specific binding motif and modeling GPCR activation. *Chemistry & biology,* 15**,** 1207-1219.

Picazo-Juarez, G., Romero-Suarez, S., Nieto-Posadas, A., Llorente, I., Jara-Oseguera, A., Briggs, M., Mcintosh, T. J., Simon, S. A., Ladron-De-Guevara, E. & Islas, L. D. 2011. Identification of a binding motif in the S5 helix that confers cholesterol sensitivity to the TRPV1 ion channel. *Journal of Biological Chemistry,* 286**,** 24966-24976.

Piskacek, M., Havelka, M., Jendruchova, K. & Knight, A. 2019. Nuclear hormone receptors: Ancient 9aaTAD and evolutionally gained NCoA activation pathways. *The Journal of steroid biochemistry and molecular biology,* 187**,** 118-123.

Reggio, P. H. 2010. Endocannabinoid binding to the cannabinoid receptors: what is known and what remains unknown. *Current medicinal chemistry,* 17**,** 1468-1486.

Reisenberg, M., Singh, P. K., Williams, G. & Doherty, P. 2012. The diacylglycerol lipases: structure, regulation and roles in and beyond endocannabinoid signalling. *Philosophical Transactions of the Royal Society B: Biological Sciences,* 367**,** 3264-3275.

Savinainen, J., Saario, S. & Laitinen, J. 2012. The serine hydrolases MAGL, ABHD6 and ABHD12 as guardians of 2‐arachidonoylglycerol signalling through cannabinoid receptors. *Acta physiologica,* 204**,** 267-276.

Sheu, S.-H., Kaya, T., Waxman, D. J. & Vajda, S. 2005. Exploring the binding site structure of the PPARγ ligand-binding domain by computational solvent mapping. *Biochemistry,* 44**,** 1193-1209.

Shim, J.-Y. 2009. Transmembrane helical domain of the cannabinoid CB1 receptor. *Biophysical journal,* 96**,** 3251-3262.

Shim, J.-Y., Bertalovitz, A. C. & Kendall, D. A. 2011. Identification of essential cannabinoid-binding domains: structural insights into early dynamic events in receptor activation. *Journal of biological chemistry,* 286**,** 33422-33435.

Sirrs, S., Van Karnebeek, C. D., Peng, X., Shyr, C., Tarailo-Graovac, M., Mandal, R., Testa, D., Dubin, D., Carbonetti, G. & Glynn, S. E. 2015. Defects in fatty acid amide hydrolase 2 in a male with neurologic and psychiatric symptoms. *Orphanet journal of rare diseases,* 10**,** 1-10.

Stadel, R., Ahn, K. H. & Kendall, D. A. 2011. The cannabinoid type‐1 receptor carboxyl‐terminus, more than just a tail. *Journal of neurochemistry,* 117**,** 1-18.

Suryanarayana, S., Daunt, D., Von Zastrow, M. & Kobilka, B. 1991. A point mutation in the seventh hydrophobic domain of the alpha 2 adrenergic receptor increases its affinity for a family of beta receptor antagonists. *Journal of Biological Chemistry,* 266**,** 15488-15492.

Uniprot 2021. UniProtKB - P51875 (GNAO_CAEEL).

Wang, C.-D., Buck, M. A. & Fraser, C. M. 1991. Site-directed mutagenesis of alpha 2A-adrenergic receptors: identification of amino acids involved in ligand binding and receptor activation by agonists. *Molecular pharmacology,* 40**,** 168-179.

Zhang, X., Yuan, Y., Wang, L., Guo, Y., Li, M., Li, C. & Pu, X. 2018. Use multiscale simulation to explore the effects of the homodimerizations between different conformation states on the activation and allosteric pathway for the μ-opioid receptor. *Physical Chemistry Chemical Physics,* 20**,** 13485-13496.

Zheng, G., Xue, W., Yang, F., Zhang, Y., Chen, Y., Yao, X. & Zhu, F. 2017. Revealing vilazodone's binding mechanism underlying its partial agonism to the 5-HT 1A receptor in the treatment of major depressive disorder. *Physical Chemistry Chemical Physics,* 19**,** 28885-28896.
