# Supplementary figures and images for: Pan-phylum In Silico Analyses of Nematode Endocannabinoid Signalling Systems Highlight Novel Opportunities for Parasite Drug Target Discovery
Source: Front Endocrinol (Lausanne). 2022 Jul 1;13:892758. doi: 10.3389/fendo.2022.892758 (PMC9283691; doi:10.3389/fendo.2022.892758)

(A)


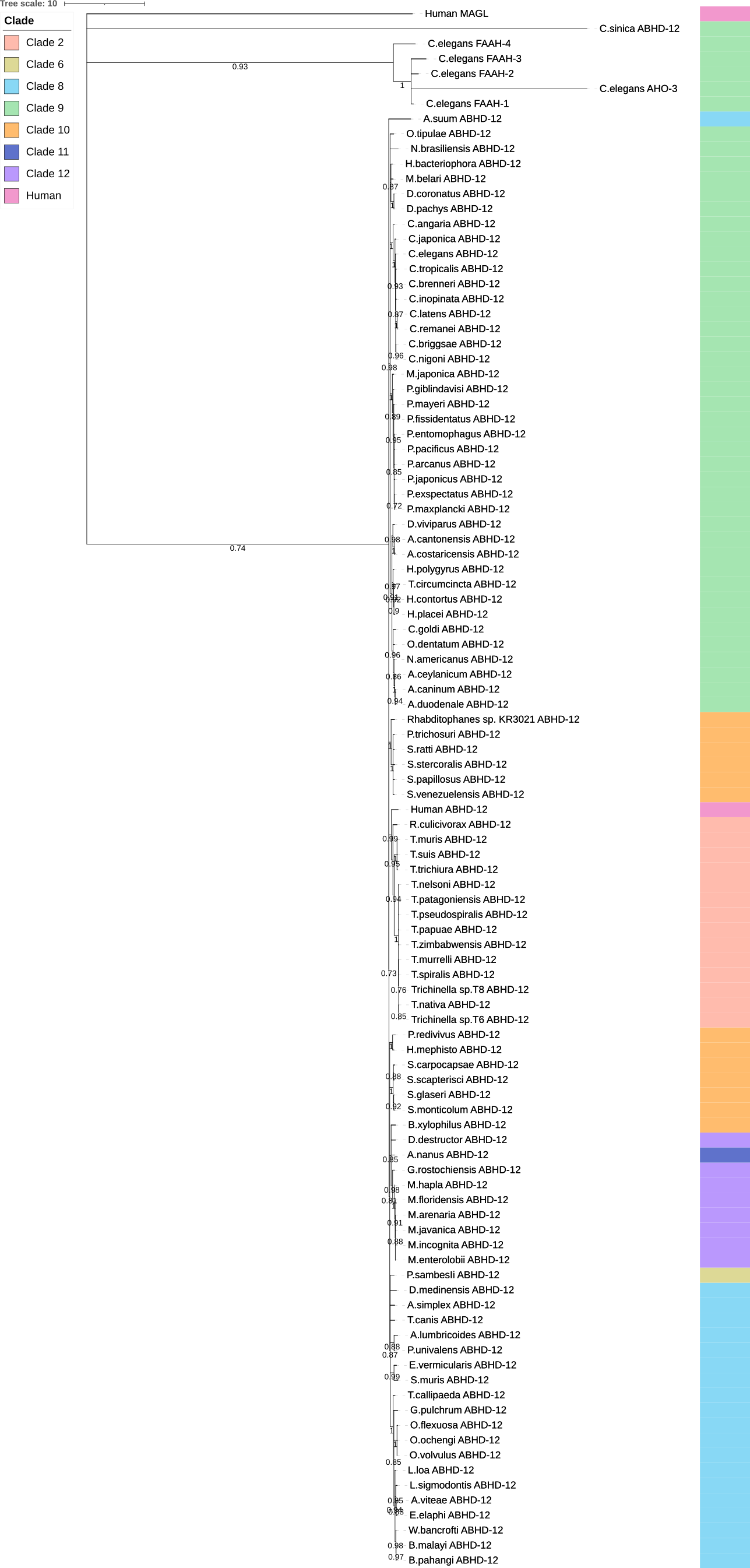


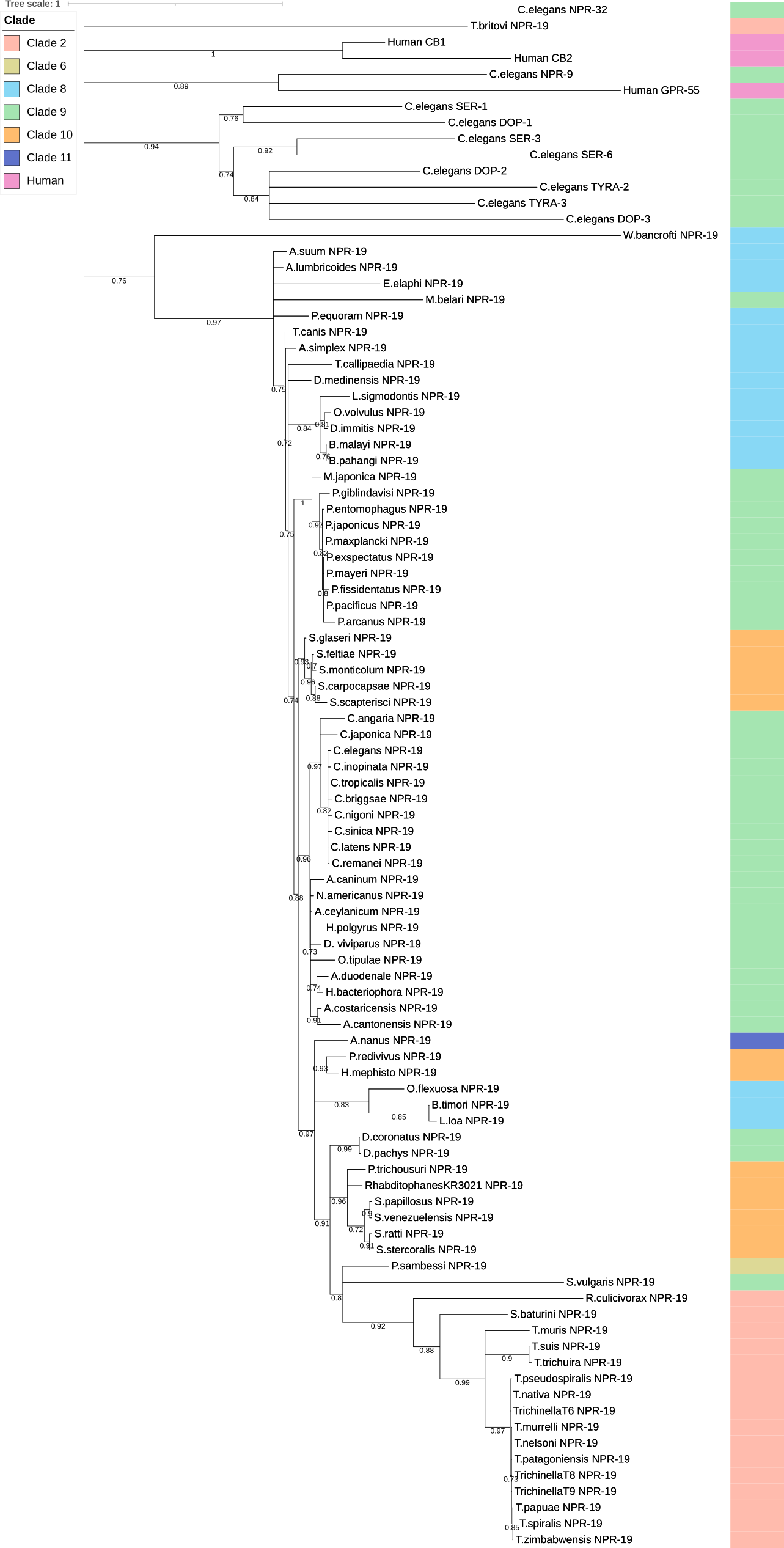


(B)


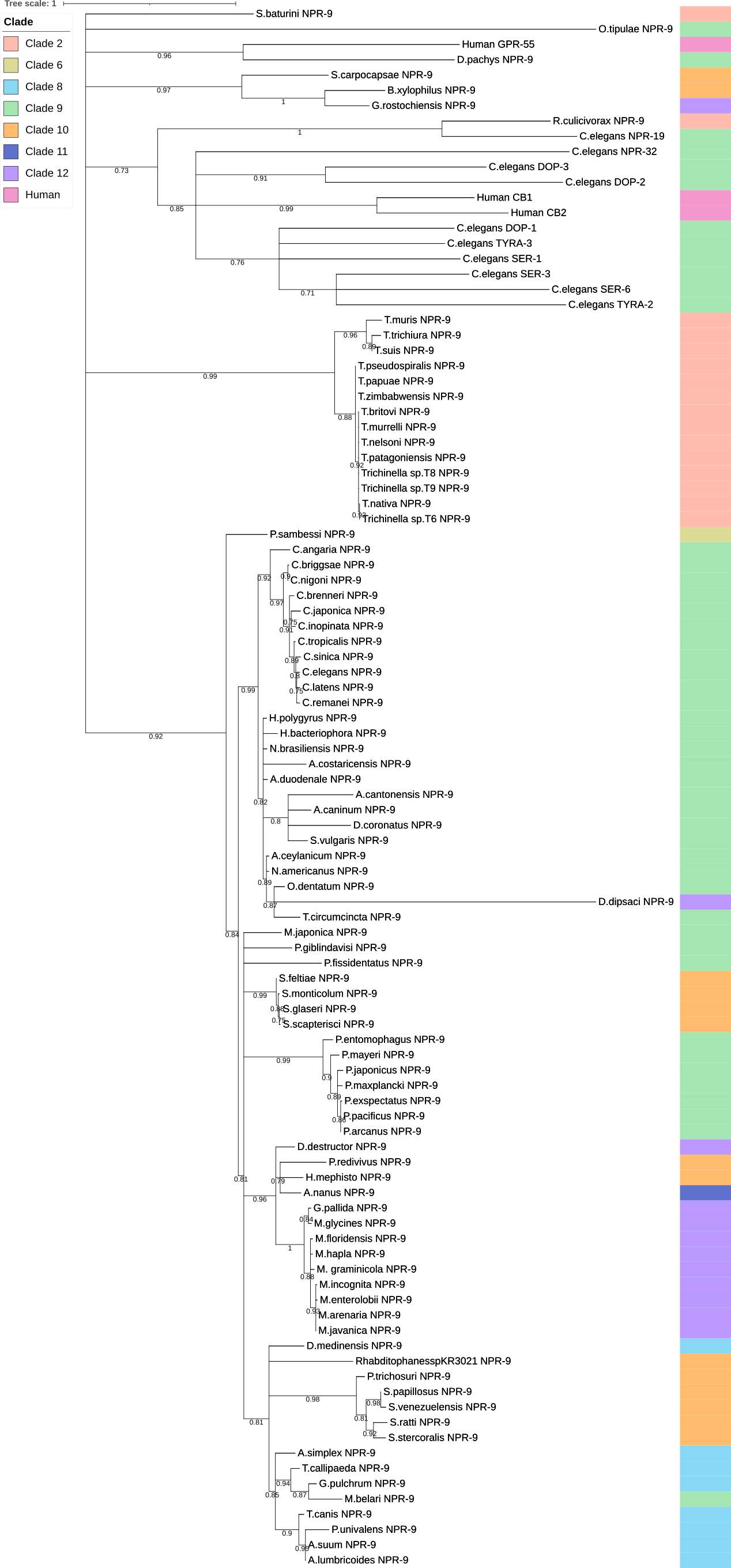


(C)


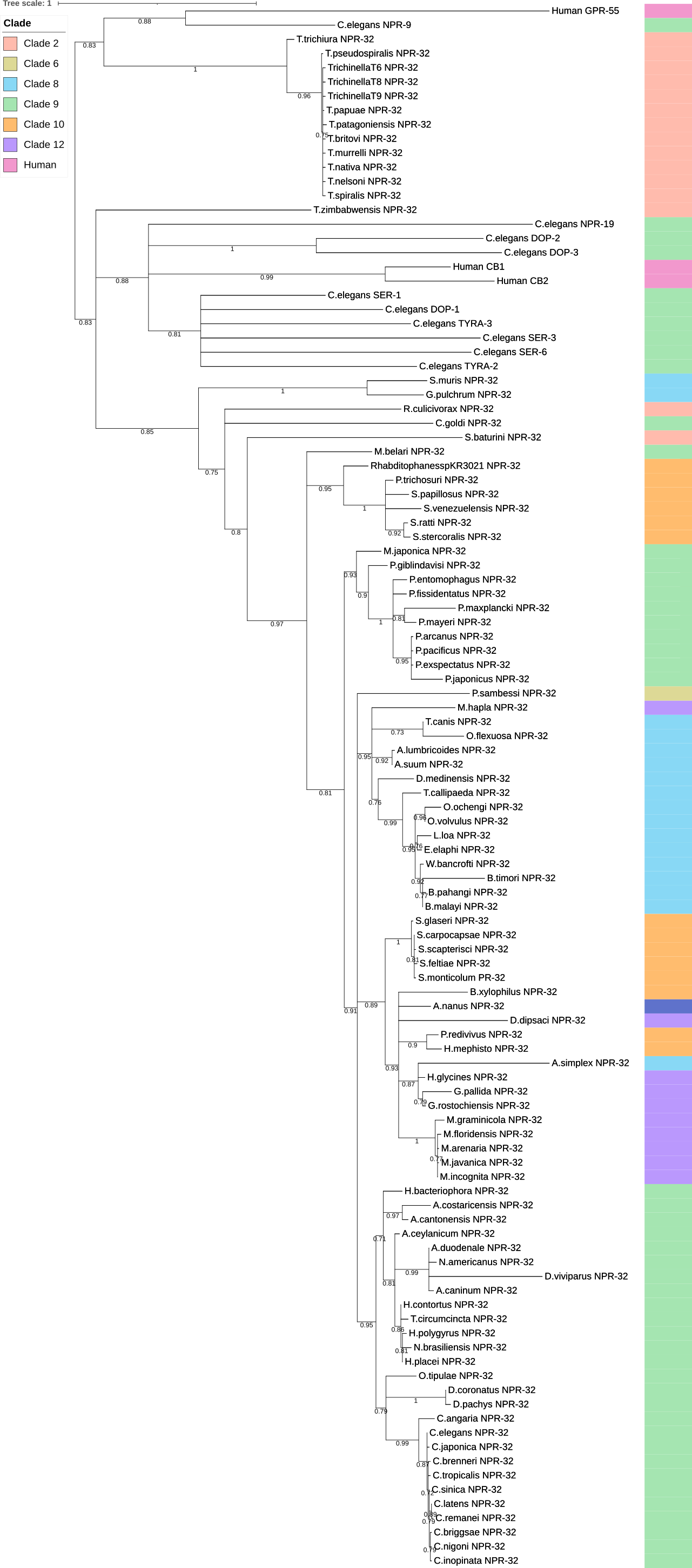


(D)


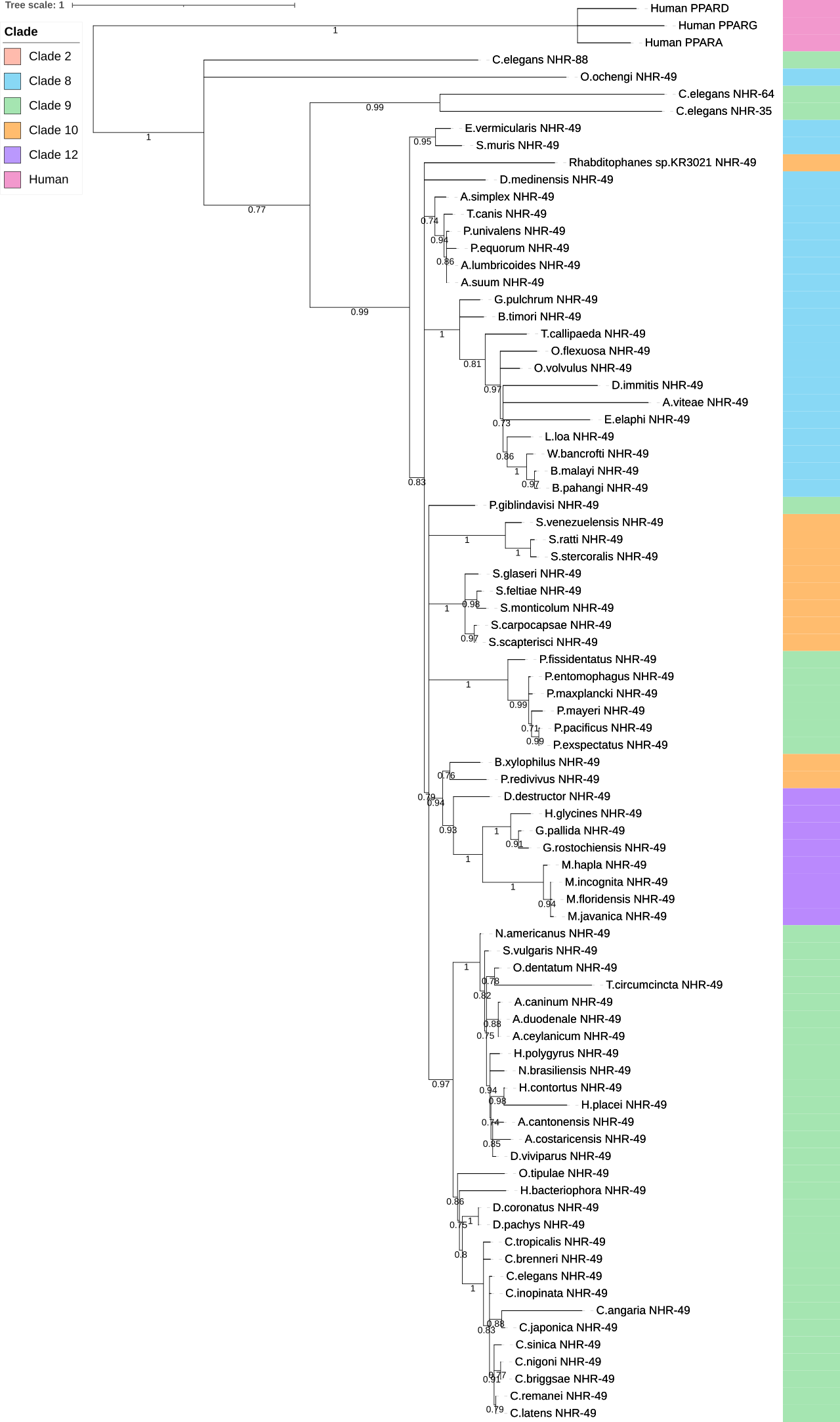


(E)


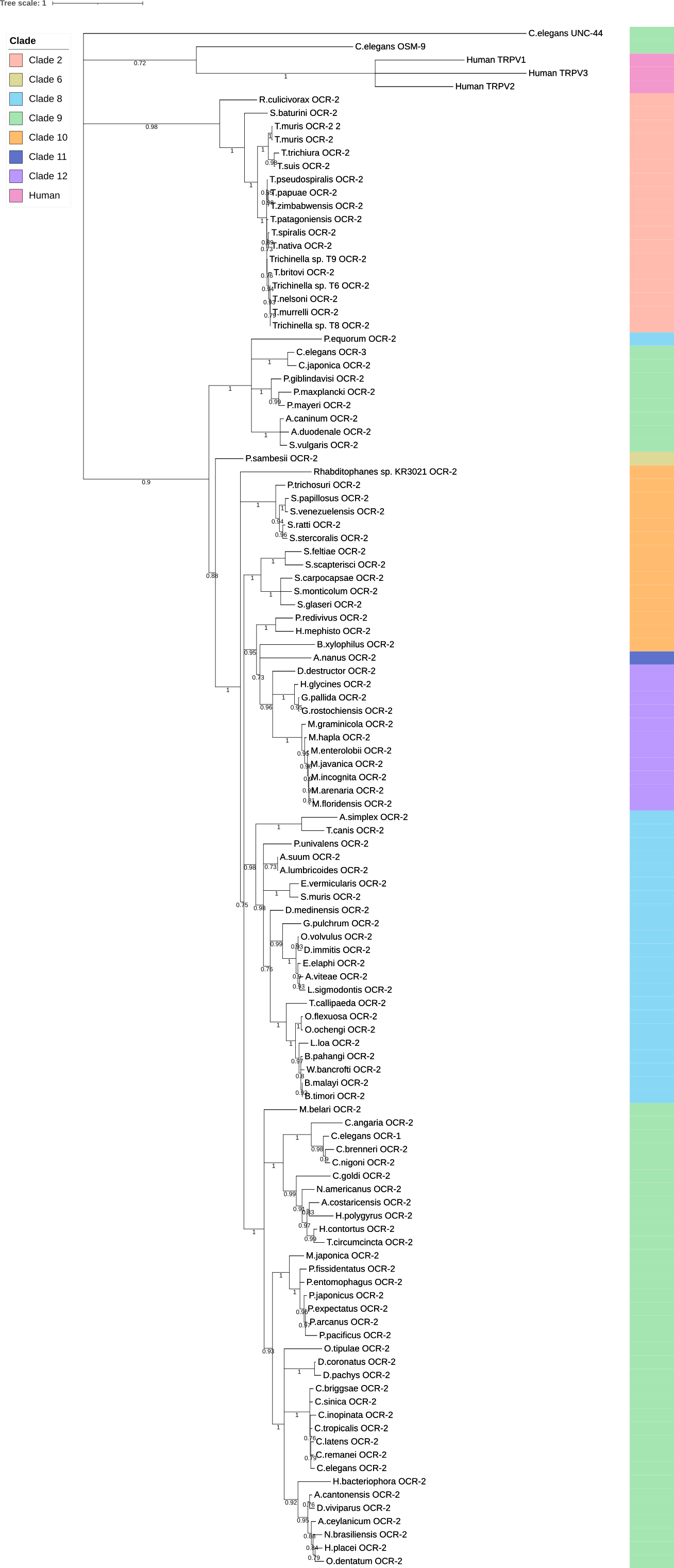


(F)


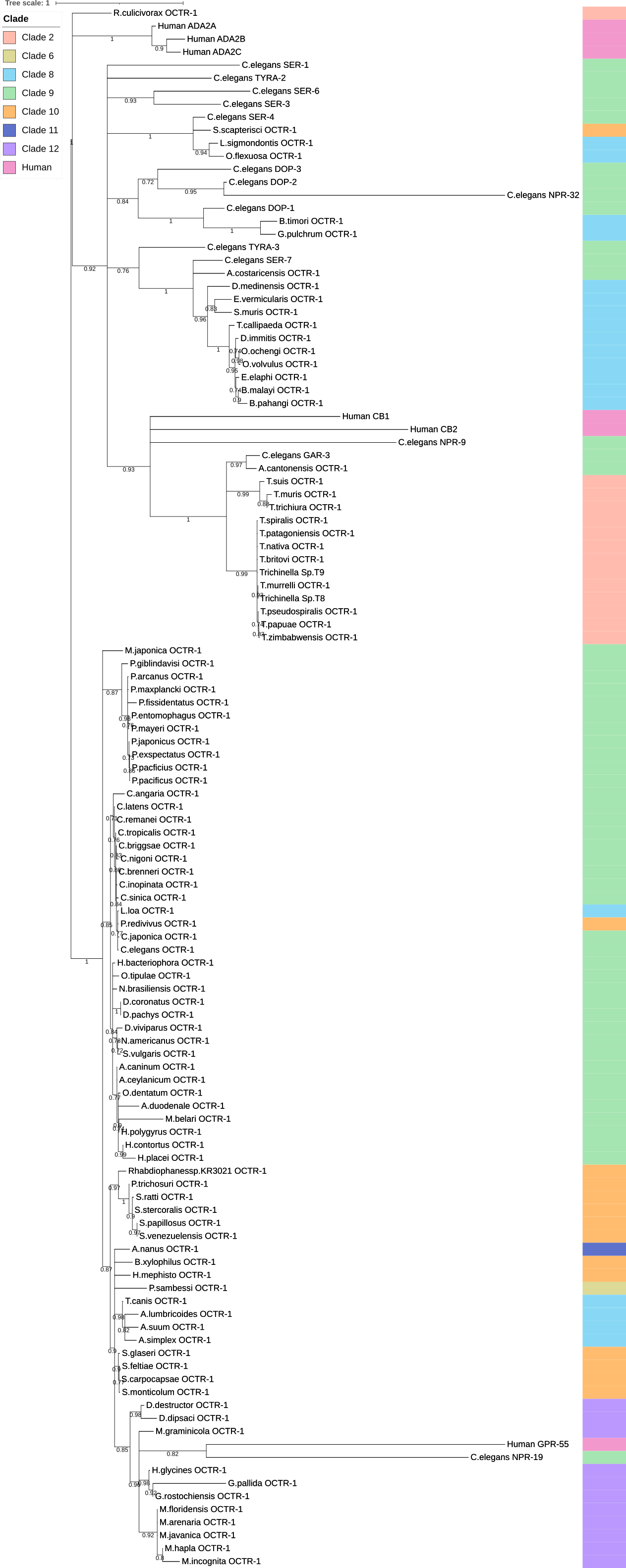


(G)


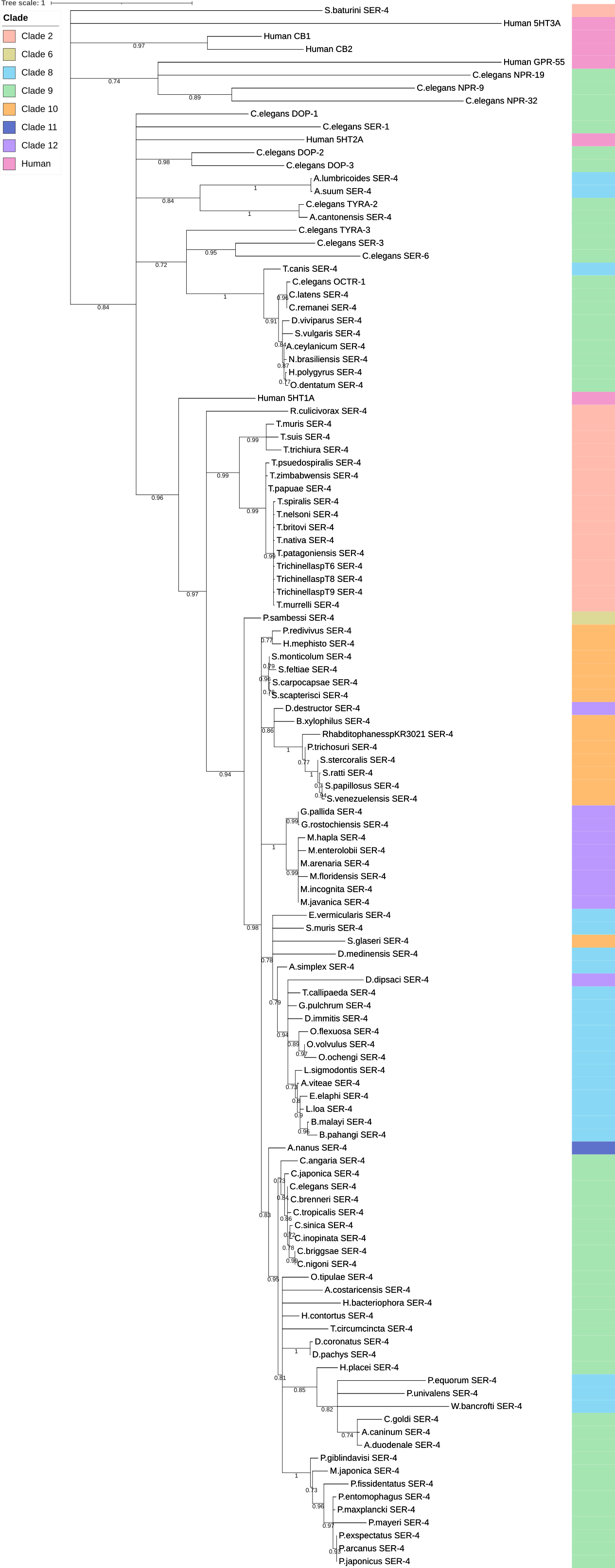


(H)

Supplement: File SI 1 — Caenorhabditis spp. EC-effector gene IDs. List of EC-effector gene IDs from Caenorhabditis spp. that were used as query sequences in this study. [file DataSheet_1.zip › Figure SI 1.DOCX]

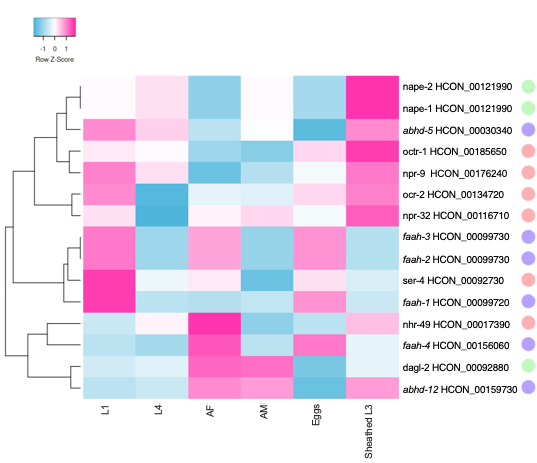

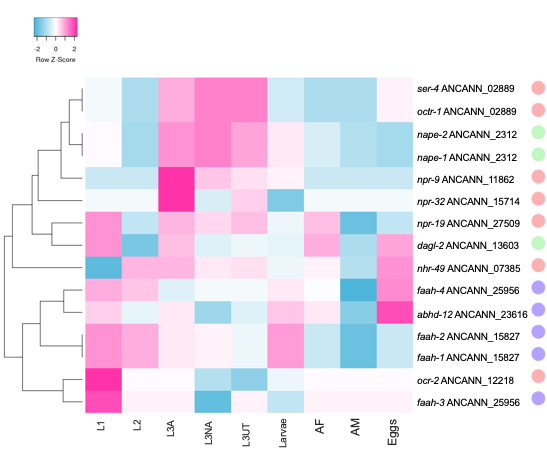


**A**

**B**

**C**

**D**

**E**

**F**


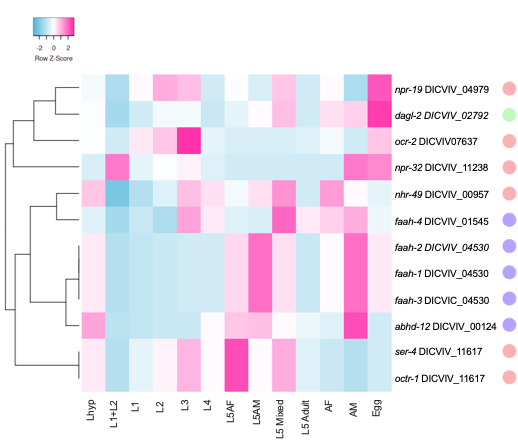

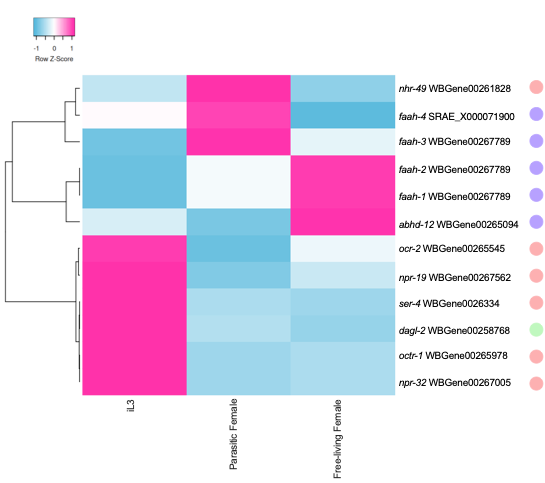

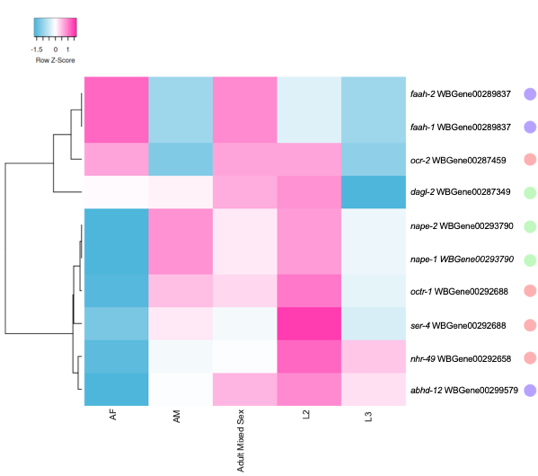


**H**

**G**

Supplement: File SI 1 — Caenorhabditis spp. EC-effector gene IDs. List of EC-effector gene IDs from Caenorhabditis spp. that were used as query sequences in this study. [file DataSheet_1.zip › Figure SI 2.DOCX]
